# Intestinal Epithelial‐Derived USP13 Alleviates Colonic Inflammation by Suppressing GRP78‐mediated Endoplasmic Reticulum Stress

**DOI:** 10.1002/advs.202500741

**Published:** 2025-07-18

**Authors:** Chenchen Qian, Chenghong Hu, Yong Xu, Wentao Xu, Zhe Wang, Wan Gan, Weiyang Tang, Lijiang Huang, Aleksandr V. Samorodov, Yi Wang

**Affiliations:** ^1^ School of Pharmacy Hangzhou Normal University Hangzhou Zhejiang 311121 China; ^2^ Chemical Biology Research Center School of Pharmaceutical Sciences Wenzhou Medical University Wenzhou Zhejiang 325035 China; ^3^ School of Medicine Hangzhou Normal University Hangzhou Zhejiang 311121 China; ^4^ Joint Research Center on Medicine the Affiliated Xiangshan Hospital of Wenzhou Medical University Ningbo Zhejiang 315700 China; ^5^ Department of Pharmacology Bashkir State Medical University Ufa 450005 Russia

**Keywords:** Deubiquitinase, ER stress, GRP78, inflammatory bowel disease, USP13

## Abstract

Inflammatory bowel disease (IBD) is a chronic condition in which endoplasmic reticulum (ER) stress and subsequent apoptosis of intestinal epithelial cells play significant roles. E3 ubiquitin ligases and deubiquitinases are crucial in IBD pathophysiology by modulating protein ubiquitination. This study investigated the regulatory role of a deubiquitinase ubiquitin‐specific peptidase 13 (USP13) in a DSS‐induced colitis mouse model and explored the underlying mechanism. Intestinal epithelial‐specific *Usp13* knockout (USP13^IEKO^) mice were treated with DSS to induce colitis. Adeno‐associated virus serotype 9 (AAV9) was constructed to overexpress UPS13 in colonic tissues. The results show that intestinal epithelial‐specific USP13 knockout exacerbated DSS‐induced colitis, linked to increased ER stress and apoptosis. Mechanistically, USP13 interacted with  GRP78, attenuating ER stress‐induced apoptosis and maintaining intestinal barrier integrity by selectively removing K63‐linked ubiquitin at lysine 327. Moreover, restoration of USP13 expression via AAV9 in USP13^IEKO^ mice attenuated DSS‐induced colitis and preserved intestinal barrier integrity. Also, USP13 levels were reduced in ulcerative colitis patients compared to the healthy controls, highlighting its protective role in intestinal health. These findings underscore the protective role of the USP13‐GRP78 axis in IBD and suggest that targeting USP13 through intestinal epithelial‐specific gene therapy may be a potential therapeutic strategy for IBD.

## Graphical abstract

USP13 deficiency in intestinal epithelial cells aggravates DSS‐induced colitis by enhancing ER stress and apoptosis. USP13 interacts with GRP78 and reduces its K63‐linked ubiquitination to alleviate epithelial injury. Restoring USP13 expression or targeting the USP13‐GRP78 axis may represent a promising strategy for treating inflammatory bowel disease by preserving mucosal barrier integrity.

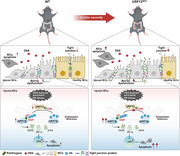

## 1 Introduction

Inflammatory bowel disease (IBD), including Crohn's disease (CD) and ulcerative colitis (UC), is a complex, multifactorial disease characterized by chronic and relapsing inflammation of the gastrointestinal tract.^[^ ^1^ ^]^ Recent evidence suggests that damage in the intestinal mucosal barrier is a key factor in IBD pathogenesis, with endoplasmic reticulum (ER) stress and subsequent apoptosis of intestinal epithelial cells (IECs) playing a crucial role.^[^ ^2^, ^3^ ^]^ Both colitis mouse models and active IBD patients often exhibit significant ER stress and apoptosis in the ileal and/or colonic epithelial tissues, leading to characteristic crypt microabscesses and increased intestinal permeability.^[^ ^4^, ^5^ ^]^ This excessive apoptosis in IECs impairs the epithelial barrier, allowing intestinal contents and microbes to translocate into the lamina propria, triggering inflammation and further epithelial injury.^[^ ^6^ ^]^ Therefore, targeting ER stress and apoptosis in IECs to restore epithelial barrier integrity presents a novel and promising therapeutic strategy for IBD.

Ubiquitination is crucial for post‐translational modifications in various inflammatory diseases, including IBD.^[^ ^7^, ^8^ ^]^ E3 ubiquitin ligases (E3s)‐mediated ubiquitination and deubiquitinases (DUBs)‐mediated deubiquitination of substrate proteins are reversible processes regulating cellular activities such as signal transduction, autophagy, and apoptosis.^[^ ^8^, ^9^ ^]^ In colonic inflammation, several DUBs influence intestinal homeostasis and injury by modulating signal transduction and protein assembly.^[^ ^7^, ^10^ ^]^ For instance, Karatzas et al. showed that intestinal epithelial cylindromatosis (CYLD) suppresses tumors in colitis‐associated carcinogenesis.^[^ ^11^ ^]^ Another DUB, tumor necrosis factor (TNF)‐α‐induced protein 3 (TNFAIP3, also known as A20), enhances TNF‐induced mucosal erosion and RIPK1‐dependent IECs apoptosis through Ripoptosome/receptor‐interacting serine/threonine‐protein kinase 1 (RIPK1) activation.^[^ ^12^ ^]^ Additionally, ubiquitin‐specific protease 38 (USP38) negatively regulates the recruitment of nuclear factor kappa B (NF‐κB) transcription factor to *interleukin 6* and *interleukin 23* promoters.^[^ ^13^ ^]^ Thus, DUBs are crucial for the immunomodulatory functions of IECs in the gut.

Ubiquitin‐specific peptidase 13 (USP13), a member of the USP family, contains a Zinc finger domain and a USP catalytic domain.^[^ ^14^ ^]^ As a DUB, USP13 regulates inflammatory homeostasis and the progression of various diseases by modulating the ubiquitination of target proteins, thereby mediating signal transduction. Previous studies have shown that USP13 can deubiquitinate and maintain signal transducer and activator of transcription 1 (STAT1) signaling, triggering the interferon (IFN) signaling cascade.^[^ ^15^ ^]^ Additionally, USP13 can inhibit the recruitment of downstream TANK‐binding kinase 1 (TBK1) by removing K27 and K33‐linked polyubiquitination from the stimulator of interferon genes (STING).^[^ ^16^ ^]^ Notably, research indicates that the USP13 transcript level increases in response to lipopolysaccharide stimulation or *enteropathogenic Escherichia coli* infection.^[^ ^7^ ^]^ All these studies indicate the potential role of USP13 in inflammatory diseases. However, whether USP13 regulates the pathogenesis of UC remains unclear, and its physiological function and underlying mechanism in maintaining intestinal homeostasis have yet to be elucidated.

In this study, we explored the effect of intestinal epithelial‐derived USP13 on dextran sulfate sodium (DSS)‐induced colitis using intestinal epithelial‐specific USP13 knockout mice and AAV9‐mediated USP13 overexpression mice. Mechanistically, we identified glucose‐regulated protein 78 (GRP78) as a crucial substrate of USP13 through interactome analysis in IECs. Our findings demonstrate the protective role of the intestinal epithelial cell‐specific USP13‐GRP78 axis in IBD, suggesting that targeting USP13 for intestinal epithelial cell‐specific gene therapy may be a potential treatment strategy for intestinal inflammatory diseases.

## 2 Result

### 2.1 Identification of Epithelial‐Derived USP13 as a Vital Factor in UC

**Figure 1:**
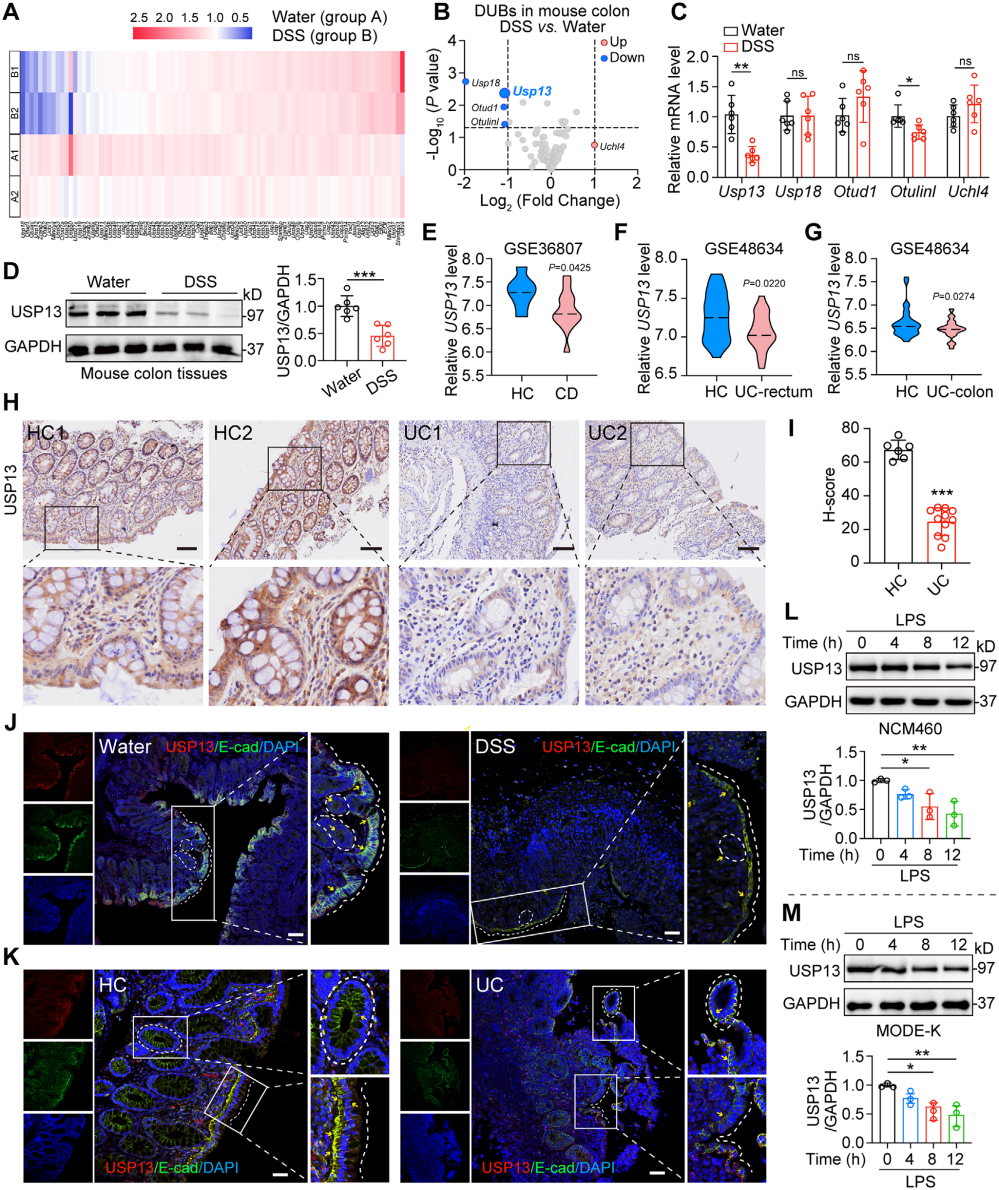
Identification of epithelial‐derived USP13 as a vital factor in ulcerative colitis. A,B) Transcriptome sequencing analysis of DUB genes in colon tissues from WT mice treated with or without 2.5% DSS. A) Heatmap showing expression changes of DUB genes in colon tissues. B) mRNA profiles of five significantly altered DUBs in colon tissues (|Fold Change| > 2, *p* < 0.05). Red and blue points represent up‐regulated and down‐regulated DUBs compared to the water‐treated group, respectively, while gray points represent DUBs with no statistical difference compared to the water‐treated group. C) Real‐time qPCR analysis of the mRNA expression of partial DUBs in colon tissues from WT mice treated with or without 2.5% DSS (*n* = 6). D) Immunoblots of USP13 protein in colon tissues from WT mice treated with or without 2.5% DSS. GAPDH was used as the loading control. Representative blots and densitometric quantification are shown (*n* = 6). E–G) The mRNA levels of *USP13* in mucosal tissues from Crohn's disease (CD) or ulcerative colitis (UC) patients and healthy controls (HC) from public datasets. E) Colonic mucosa from CD (*n* = 13) and HC (*n* = 7) patients (GSE36807). F) Rectal tissues from UC (*n* = 21) and HC (*n* = 25) patients (GSE48634). G) Colonic mucosa from UC (*n* = 32) and HC (*n* = 41) patients (GSE48634). H) Representative immunohistochemical staining of USP13 in the colon tissues of patients with UC and HC. Brown indicates immune‐positive staining, and blue represents nuclear counterstaining (scale bar: 100 µm). I) Quantification of USP13 expression based on H‐score analysis (UC, *n* = 11; HC, *n* = 6). J,K) The cellular localization of USP13 in colon tissues was determined by immunofluorescence staining (scale bar: 100 µm). USP13 (red), E‐cadherin (green), and DAPI (nuclei, blue) were visualized in mouse colon tissues J) and in colonic tissues from UC patients and HC (K). L,M) Immunoblots of USP13 protein in NCM460 L) or MODE‐K cells M) stimulated by LPS (5 µg mL^−1^, different time points). GAPDH was used as the loading control. Representative blots and densitometric quantification are shown (*n* = 3). Data are presented as mean ± SD; ^*^ *p* < 0.05; ^**^ *p* < 0.01; ^***^ *p* < 0.001; ns = not significant.

We first analyzed the mRNA profile of DUBs in DSS‐induced mouse colitis through transcriptome sequencing (**Figure** **1**A). Five DUBs (*Usp13*, *Usp18*, *Otud1*, *Otulinl*, and *Uchl4*) showed significant changes (|Fold Change| > 2, *p *< 0.05) compared with the control tissues (Figure 1B). We then confirmed the transcriptional changes of these five DUBs by measuring the mRNA levels in DSS‐challenged mouse colon tissues. Our data indicated that the mRNA levels of *Usp13* and *Otulinl* showed a significant decrease, especially for *Usp13* (Figure 1C). At the same time, mRNA levels of *Usp18*, *Otud1*, and *Uchl4* did not markedly change (Figure 1C). Although *Usp18* initially showed the most significant change in the transcriptome data, subsequent validation experiments (e.g., qPCR) indicated that *Usp13* exhibited more consistent and stable changes across different experimental conditions. These data indicated the substantial research significance of *Usp13*.

Western blotting analysis further confirmed that USP13 protein expression was significantly reduced in the colon tissues of DSS‐treated mice (Figure 1D). Using publicly available datasets from the Gene Expression Omnibus (GEO), we examined USP13 expression in inflamed intestinal mucosa from Crohn's disease (CD) patients. The results revealed a marked reduction in USP13 expression compared to healthy controls (HC) (Figure 1E). We then extended the analysis to ulcerative colitis (UC), which typically originates in the rectum and spreads proximally along the colon. Similarly, USP13 expression was significantly decreased in inflamed rectal and colonic tissues from UC patients, suggesting a potential role of USP13 in maintaining epithelial homeostasis during IBD‐associated inflammation (Figure 1F,G). Moreover, immunohistochemical staining of colonic biopsy samples revealed reduced USP13 protein levels in UC patients, with USP13 predominantly localized in intestinal epithelial cells (IECs) (Figure 1H,I). Double immunofluorescence staining further confirmed that downregulation of USP13 was mainly observed in E‐cadherin⁺ IECs in both DSS‐induced mice and UC patient tissues (Figure 1J,K).

To further explore USP13 regulation under inflammatory conditions in vitro, we used a well‐established model in which IECs are stimulated with lipopolysaccharide (LPS) to mimic colitis‐associated inflammation. Previous studies have shown that LPS stimulation of IECs is widely used to investigate inflammatory mechanisms in colitis models.^[^ ^17^, ^18^, ^19^, ^20^ ^]^ Consistently, LPS stimulation led to a time‐dependent reduction of USP13 protein expression in both NCM460 (Figure 1L) and MODE‐K cells (Figure 1M). Collectively, these results suggest that USP13 in IECs may play a role in the pathogenesis of UC.

### 2.2 Intestinal Epithelial‐Specific USP13 Deficiency Aggravates DSS‐Induced Colitis In Vivo

**Figure 2:**
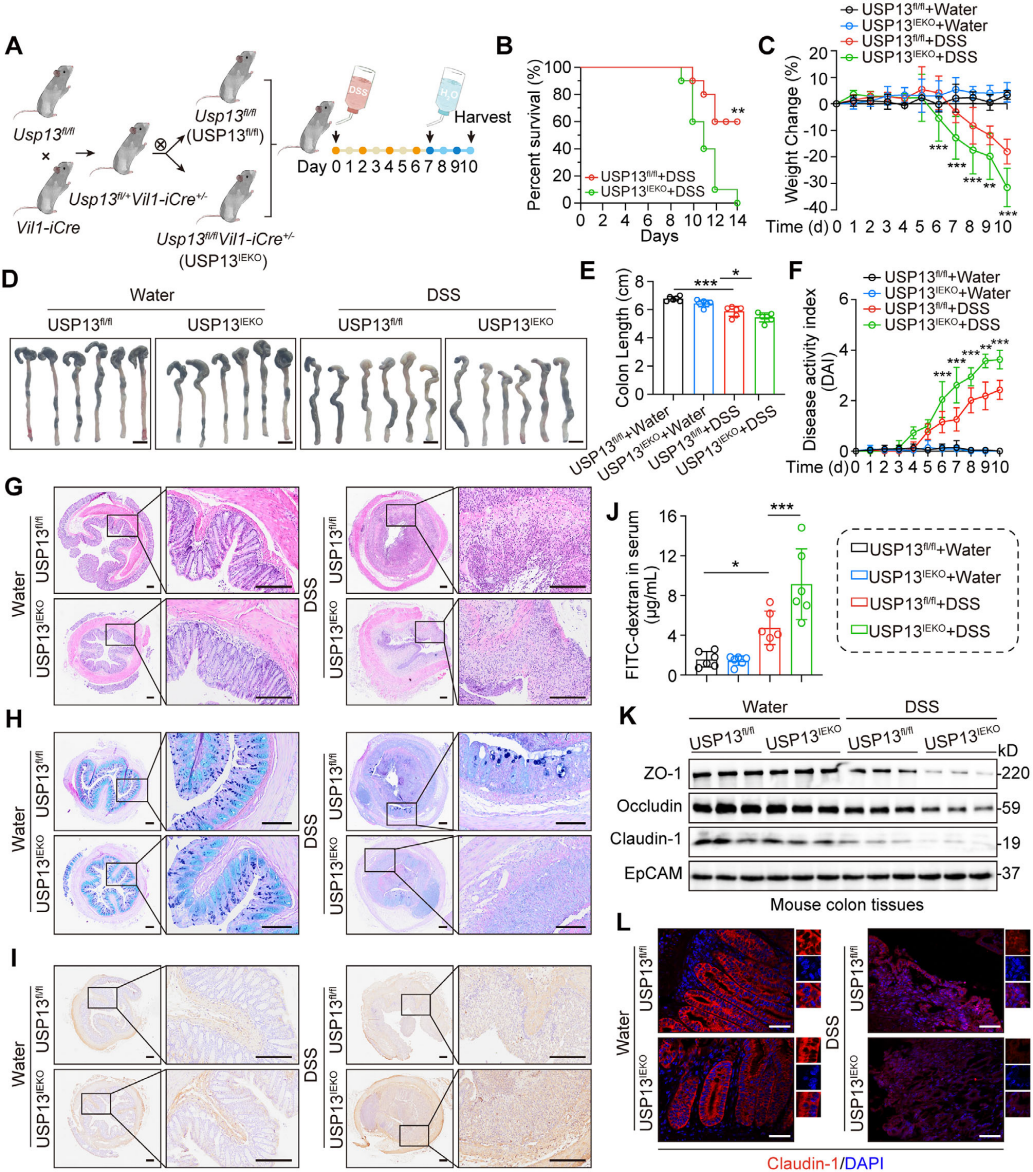
Intestinal epithelial‐specific USP13 deficiency aggravates DSS‐induced mouse colitis. A) Schematic strategy for generating intestinal epithelial‐specific USP13 knockout (USP13^IEKO^) mice and the experimental timeline of the DSS‐induced colitis. USP13^IEKO^ and USP13^fl/fl^ mice were given 2.5% DSS in drinking water for 7 days, followed by 3 days of regular water. B) Kaplan‐Meier survival curves of USP13^IEKO^ and USP13^fl/fl^ mice treated with DSS‐induced colitis for 14 days (*n* = 10). The log‐rank test was used to calculate the *p*‐value for survival percentage. C) Weight change in USP13^IEKO^ and USP13^fl/fl^ mice with DSS‐induced colitis (*n* = 6). D,E) Gross morphology D) and the colon length E) of colon tissues from USP13^IEKO^ and USP13^fl/fl^ mice on day 10 after DSS initiation (scale bar: 1 cm). F–I) Disease activity index F), representative H&E staining G), AB‐PAS staining H), and Ki67 staining I) of colon tissues from USP13^IEKO^ and USP13^fl/fl^ mice on day 10 after DSS initiation (*n* = 6‐7, scale bar: 200 µm). J) Intestinal permeability was assessed by FITC‐dextran assays on day 10 after DSS initiation (*n* = 6). K) Representative immunoblots of ZO‐1, Occludin, and Claudin‐1 proteins in colon tissues on day 10 after DSS initiation. EpCAM was used as the loading control. L) Representative immunofluorescence of Claudin‐1 (red) and DAPI (nuclei, blue) in colon tissues on day 10 after DSS initiation (scale bar: 100 µm). Data are presented as mean ± SD; ^*^ *p* < 0.05; ^**^ *p* < 0.01; ^***^ *p* < 0.001.

Building upon our previous identification of epithelial‐derived USP13 as a vital factor in UC, we further investigated its role using an intestinal epithelial‐specific *Usp13* deletion mouse model (*Vil‐iCre*; *Usp13^fl/fl^*, hereafter termed USP13^IEKO^; **Figure** **2**A; Figure S1A, Supporting Information). USP13^IEKO^ mice and littermate USP13^fl/fl^ mice (used as the control counterpart) were subjected to 2.5% DSS in water continuously for 7 days, followed by 3 days of recovery (Figure 2A). Intestinal epithelial USP13 deficiency significantly reduced the survival rate of colitis mice after 2.5% DSS challenge (Figure 2B). USP13^IEKO^ mice exhibited more severe weight loss (Figure 2C) and increased colon shortening (Figure 2D,E), along with higher DAI scores compared to the USP13^fl/fl^ mice treated with DSS (Figure 2F). Further histopathological examination revealed that USP13^IEKO^ mice exhibited a more severe disruption of the colonic epithelial barrier and more pronounced ulceration compared to those of USP13^fl/fl^ mice following DSS challenge (Figure 2G; Figure S2A, Supporting Information). AB‐PAS staining also indicated a higher reduction of goblet cells in USP13^IEKO^ mice than in USP13^fl/fl^ mice after DSS treatment (Figure 2H). Ki67 staining demonstrated that intestinal epithelial USP13 deficiency increased DSS‐induced cell proliferation in colon tissues (Figure 2I; Figure S1B, Supporting Information).

The extracellular mucus and tight junctions are crucial elements of the intestinal mucosal barrier, essential for shielding against harmful substances within the intestinal lumen.^[^ ^21^, ^22^ ^]^ Damage to intestinal permeability may stem from dysregulation of the mucus or the epithelial junctional complex, heightening vulnerability to colitis.^[^ ^21^, ^22^ ^]^ Changes in epithelial permeability were measured using the FITC‐dextran experiment. Our data show that USP13^IEKO^ mice exhibited significantly reduced colonic mucosal permeability when compared to USP13^fl/fl^ mice (Figure 2J). Consistently, we found that USP13^IEKO^ mice significantly decreased the levels of tight junction proteins ZO‐1, Occludin, and Claudin‐1 at both mRNA and protein levels compared to USP13^fl/fl^ mice treated with DSS (Figure 2K; Figure S1C–H, Supporting Information), indicating that intestinal epithelial‐specific USP13 deficiency exacerbates epithelial permeability in DSS‐induced colitis and disturbs intestinal homeostasis. Furthermore, the immunofluorescence results of Claudin‐1 further confirmed the exacerbating effect of intestinal epithelial‐specific USP13 deficiency on DSS‐induced colitis (Figure 2L). Taken together, these findings underscore the crucial role of intestinal epithelial‐specific USP13 deficiency in exacerbating DSS‐induced colitis by compromising epithelial permeability and aggravating colonic injury, thus increasing susceptibility to DSS‐induced colitis.

### 2.3 Intestinal Epithelial‐Specific USP13 Deficiency Increased ER Stress and Increased Apoptosis in Mice with DSS‐Induced Colitis

**Figure 3:**
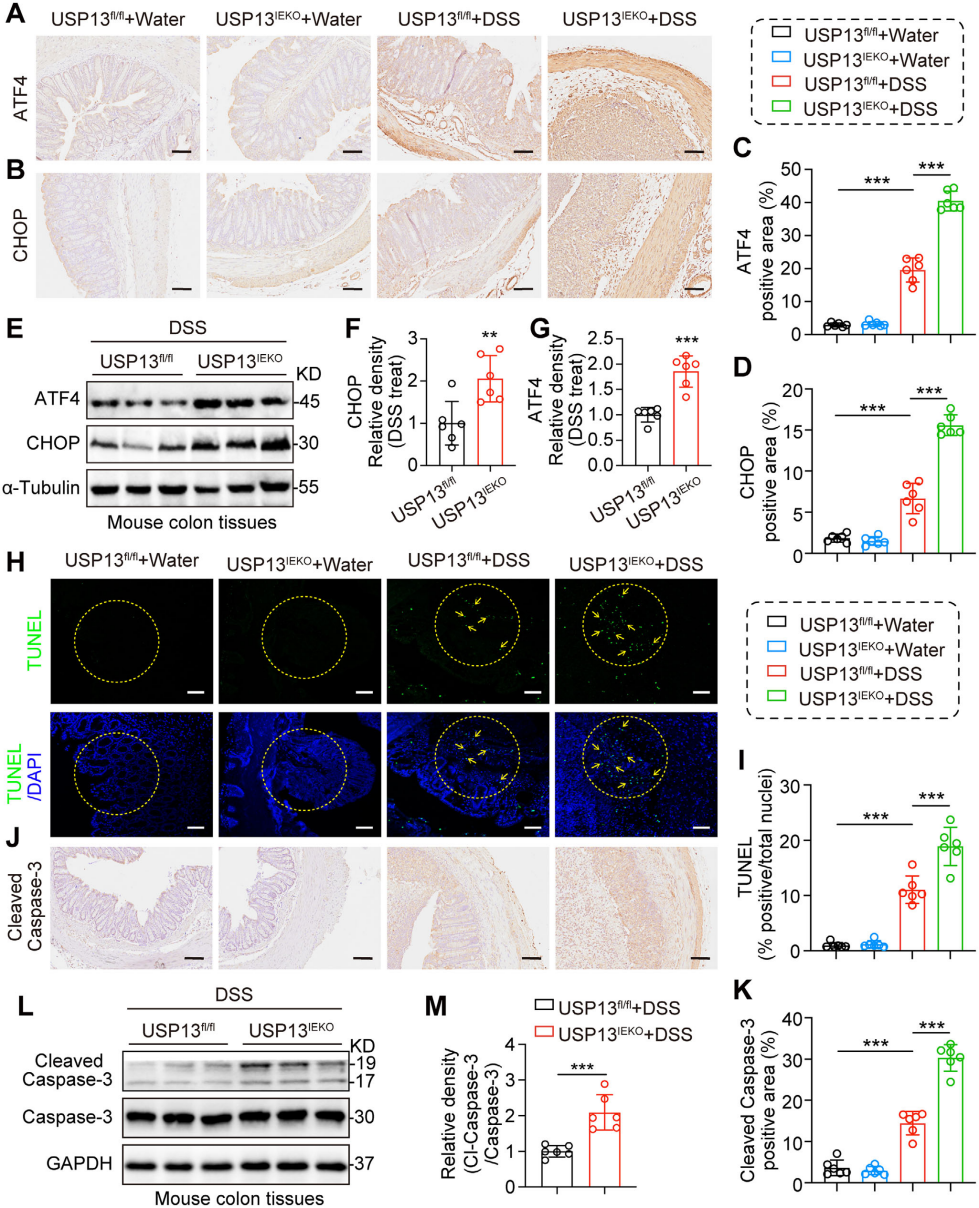
Intestinal epithelial‐specific USP13 deficiency increased ER stress and increased apoptosis in colon tissues of DSS‐treated mice. A–D) Representative IHC images of ATF4 A) and CHOP B) in colon tissues (scale bar: 100 µm). Quantification of ATF4 C) and CHOP D) was shown (*n* = 6). E–G) Representative immunoblots of ATF4 and CHOP proteins levels in colon tissues from DSS‐induced colitis mice. α‐Tubulin was used as the loading control. Representative blots E) and densitometric quantification (*n* = 6) are shown F,G). H–K) Representative images of TUNEL H), and Cleaved Caspase‐3 staining J) in colon tissues (scale bar: 100 µm). Quantification of TUNEL I), and Cleaved Caspase‐3 positive area K) was shown (*n* = 6). I,J) Representative Immunoblots of Cleaved Caspase‐3 and Caspase‐3 proteins levels in colon tissues from DSS‐induced colitis mice. GAPDH was used as the loading control. Representative blots L) and densitometric quantification (*n* = 6) are shown M). Data are presented as mean ± SD; ^**^ *p* < 0.01; ^***^ *p* < 0.001.

Recent studies suggest that unresolved epithelial ER stress may contribute to the pathogenesis of UC by disrupting protein secretion, inducing epithelial cell apoptosis, and triggering intestinal inflammation.^[^ ^23^, ^24^ ^]^ Thus, we further investigated the role of intestinal epithelial‐specific USP13 deficiency in ER stress and cell death in vivo. Following DSS treatment, USP13^IEKO^ mice exhibited higher levels of ER stress in colon tissues compared to USP13^fl/fl^ mice, as indicated by immunohistochemistry and immunoblotting of ATF4, CHOP, and increased mRNA level of *Xbp1s* (**Figure** **3**A–G; Figure S1I, Supporting Information). In contrast, no significant differences were observed between water‐treated USP13^IEKO^ and USP13^fl/fl^ mice. Additionally, DSS‐treated USP13^IEKO^ mice showed more pronounced apoptosis in colon tissues, demonstrated by TUNEL staining (Figure 3H,I), and further confirmed by immunohistochemistry and immunoblotting of Cleaved Caspase‐3 (Figure 3J,K). This may be due to an initial increase in cell proliferation caused by intestinal epithelial‐specific USP13 deficiency, which ultimately leads to elevated apoptosis as ER stress accumulates. In summary, under DSS‐induced colitis conditions, intestinal epithelial‐specific USP13 deficiency exacerbates DSS‐induced colitis by increasing ER stress and apoptosis in colon tissues.

### 2.4 USP13 Alleviates ER Stress and Apoptosis, Restoring Intestinal Barrier Integrity in Colonic Epithelial Cells

**Figure 4:**
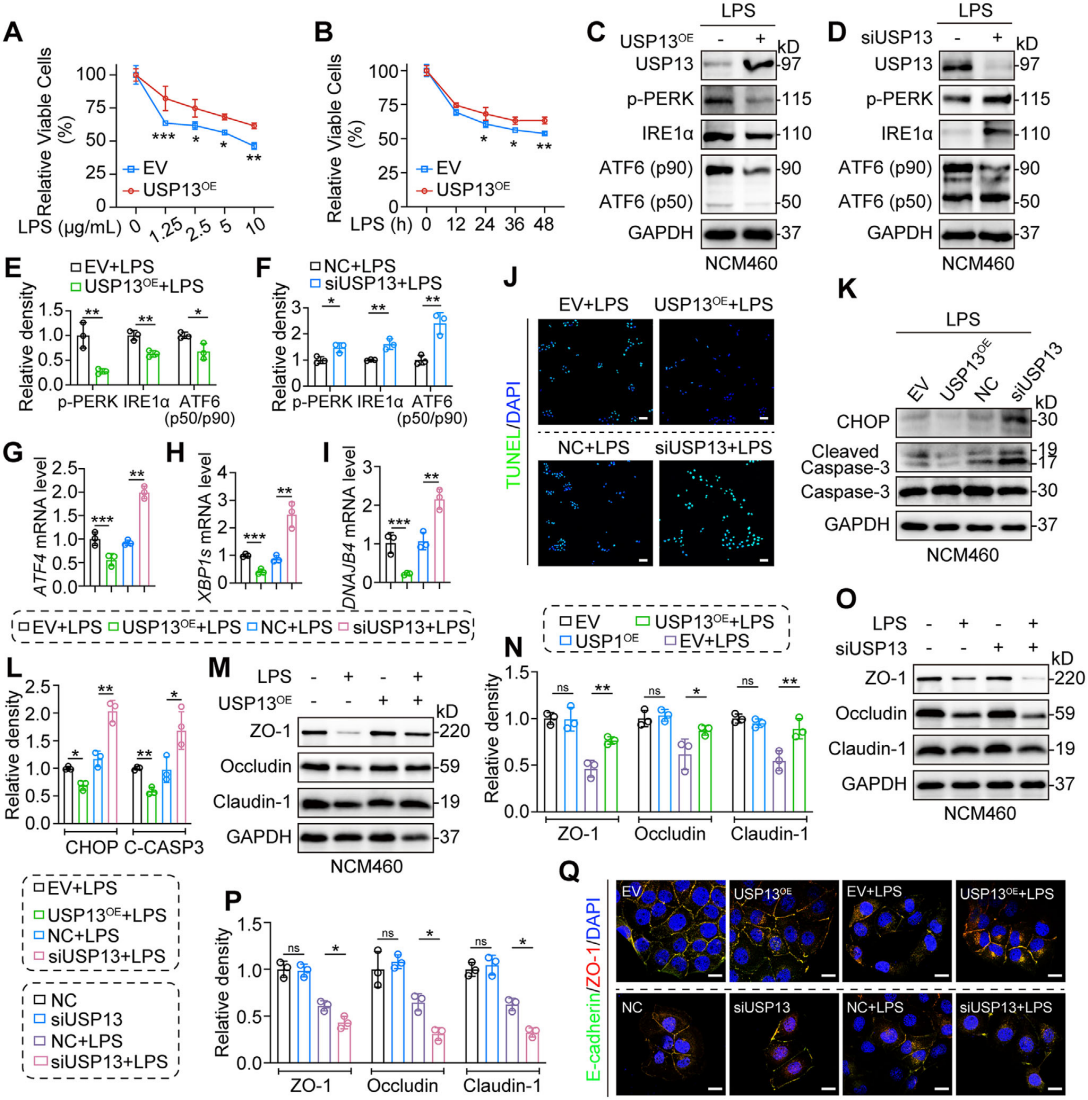
USP13 alleviates LPS‐induced ER stress and restores intestinal barrier integrity by reducing apoptosis in colonic epithelial cells. A,B) CCK‐8 assay of viable NCM460 cells overexpressing either EV or USP13^OE^ plasmids, treated with various LPS concentrations for 48 h A) or with 5 µg mL^−1^ LPS B) for different time periods (*n* = 3). C–F) Immunoblots of USP13, p‐PERK, IRE1α, and ATF6 p50/p90 proteins in control, USP13‐overexpressing, and USP13‐silenced NCM460 cells following LPS treatment (5 µg mL^−1^) for 12 h. GAPDH was used as the loading control (*n* = 3). Representative blots are shown C,D) with densitometric quantification E,F). G–I) mRNA levels of *ATF4*, *DNAJB4*, and *XBP1s* in control, USP13‐overexpressing, or USP13‐silenced NCM460 cells following LPS treatment (5 µg mL^−1^) for 12 h. Data were normalized to the levels of *Actb* (*n* = 3). J–Q) Control, USP13‐overexpressing, or USP13‐silenced NCM460 cells were treated with LPS (5 µg mL^−1^) for 24 h. J) Representative images of apoptotic cells (scale bar: 100 µm). K–L) Immunoblots of CHOP, Cleaved Caspase‐3, and Caspase‐3 proteins. GAPDH was used as the loading control (*n* = 3). Representative blots K) and densitometric quantification are shown L). M–P) Immunoblots of ZO‐1, Occludin, and Claudin‐1 proteins. GAPDH was used as the loading control (*n* = 3). Representative blots M,O) and densitometric quantification are shown N,P). Q) Representative immunofluorescence of E‐cadherin (green), ZO‐1(red), and DAPI (nuclei, blue) (scale bar: 20 µm). Data are presented as mean ± SD; ^*^ *p* < 0.05; ^**^ *p* < 0.01; ^***^ *p* < 0.001; ns = not significant.

To further investigate the function of USP13 in UC, we assessed its effect on the viability of LPS‐treated NCM460 cells. LPS stimulation of IECs is commonly used as an in vitro model to study inflammation‐related mechanisms in colitis.^[^ ^17^, ^18^, ^19^, ^20^ ^]^ Therefore, we further examined the regulatory role of USP13 in IECs under LPS‐induced inflammatory conditions. As shown in **Figure** **4**A,B, USP13 overexpression (USP13^OE^) significantly increased the viability of NCM460 cells in a concentration‐ and time‐dependent manner after LPS treatment. Similar results were observed with tunicamycin (Tm), an ER stress inducer, confirming the crucial role of USP13 in inducing ER stress in IECs (Figure S3A,B, Supporting Information). Considering the UPR signaling pathways' response to ER stress, we measured key markers related to ER stress using the Western blot assay. As expected, USP13^OE^ decreased LPS‐induced ER stress in NCM460 cells, while silencing USP13 with siRNA (siUSP13) increased LPS‐induced ER stress (Figure 4C–F). Similarly, USP13^OE^ reversed Tm‐induced ER stress in IECs, whereas siUSP13 had the opposite effect (Figure S3C,D, Supporting Information). Additionally, USP13^OE^ reduced the transcription of several ER stress‐related genes, including *ATF4*, *DNAJB4*, and *XBP1s* (Figure 4G–I), whereas siUSP13 had the opposite effect. These data suggest a critical role of USP13 in alleviating ER stress in IECs.

ER stress and UPR dysregulation can induce epithelial cell death and compromise intestinal barrier function.^[^ ^25^, ^26^ ^]^ Our data showed that USP13^OE^ reduced LPS‐induced apoptosis, as evidenced by TUNEL staining (Figure 4J) and protein levels of CHOP and Cleaved Caspase‐3 (Figure 4K,L), while siUSP13 exacerbated LPS‐induced apoptosis (Figure 4J–L). We also examined the effect of USP13 on intestinal barrier integrity. Western blot analysis indicated that USP13^OE^ significantly upregulated the protein levels of ZO‐1, Occludin, and Claudin‐1 compared to the LPS‐treated group (Figure 4M,N), suggesting that USP13^OE^ can restore the intestinal barrier. In contrast, siUSP13 inhibited the expression of these tight junction proteins (Figure 4O,P). Similarly, USP13^OE^ reversed the Tm‐induced reduction of tight junction proteins in IECs, while siUSP13 had the opposite effect (Figure S3E–H, Supporting Information). Immunofluorescence colocalization analysis of E‐cadherin and ZO‐1 further confirmed the ability of USP13^OE^ to restore the intestinal barrier and the disruptive effect of siUSP13 (Figure 4Q). In summary, USP13 alleviates ER stress induced by LPS or Tm, leading to a reduction of apoptosis and restoration of intestinal barrier integrity in IECs.

### 2.5 Identification of GRP78 as the Potential Substrate Protein of USP13 in DSS‐Induced Colitis

**Figure 5:**
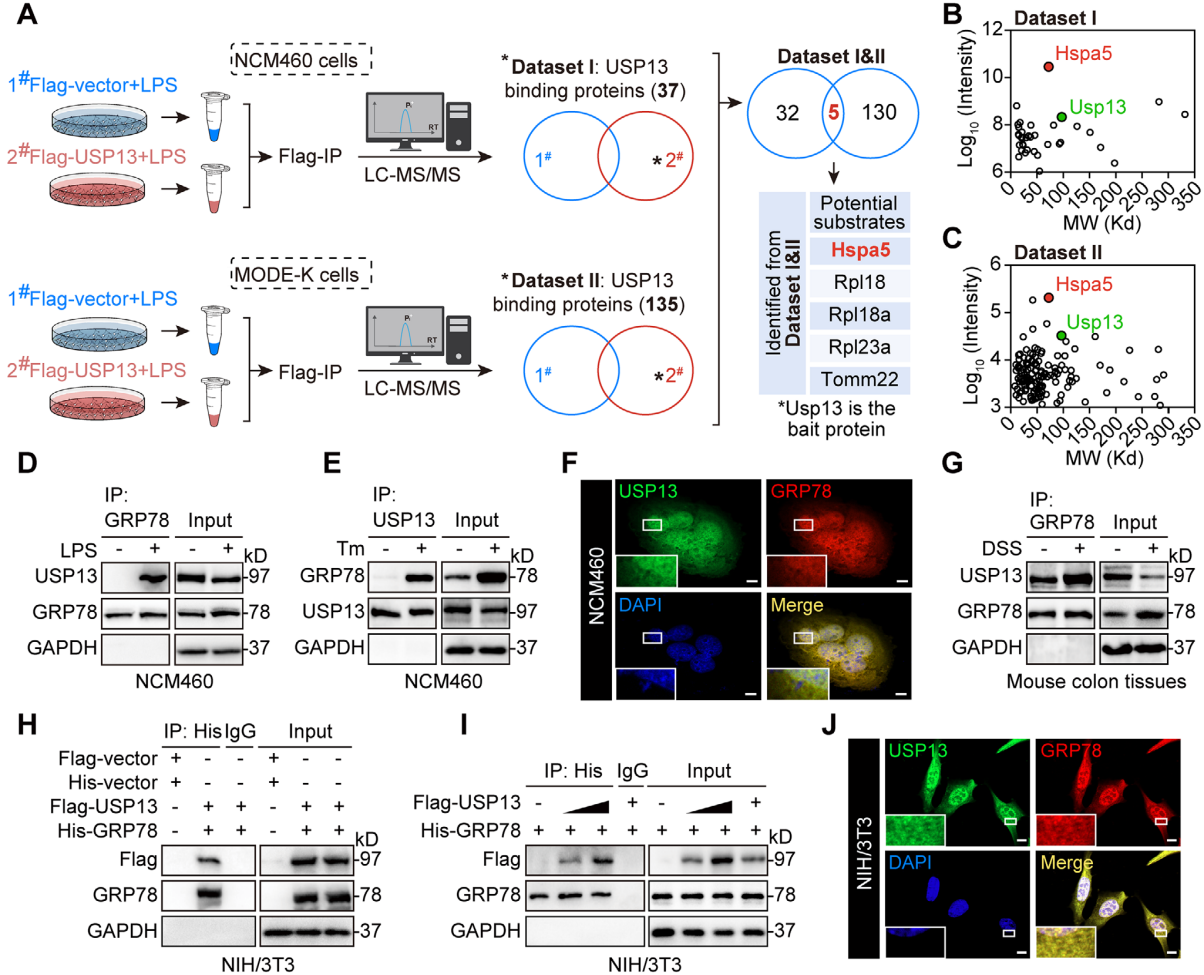
Identification of GRP78 as the potential substrate of USP13 in colonic epithelial cells. A) Schematic illustration of two interactome analyses for USP13 substrate screening. NCM460 cells (Dataset I) and MODE‐K cells (Dataset II) were transfected with Flag‐vector or Flag‐USP13 plasmids, followed by LPS stimulation. Anti‐Flag and protein G‐Sepharose beads were added to the cell lysates for co‐IP. The binding proteins were extracted, digested to peptides, and then subjected to LC‐MS/MS analysis. The following table shows the potential substrates of USP13 screened by the interactomes. B,C) 2D plots with the Log_10_ signal intensity of the quantified proteins on the *y*‐axis (revealing the enrichment in Flag‐USP13‐IP) and the molecular weight (MW) of proteins on the *x*‐axis were identified from Datasets I B) and II C). D,E) Co‐IP of endogenous USP13 and GRP78 in lysates of LPS‐stimulated D) and tunicamycin (Tm)‐stimulated E) NCM460 cells. F) Co‐localization of endogenous USP13 (green), GRP78 (red), and DAPI (nuclei, blue) in LPS‐stimulated NCM460 cells (scale bar: 10 µm). G) Co‐IP of endogenous USP13 and GRP78 in lysates of colon tissues from DSS‐treated mice. H,I) Co‐IP of exogenous Flag‐USP13 and His‐GRP78 in lysates from NIH/3T3 cells expressing Flag‐USP13 and His‐GRP78. J) Co‐localization of exogenous USP13 (green), GRP78 (red), and DAPI (nuclei, blue) in NIH/3T3 cells (scale bar: 10 µm).

DUBs perform their specific functions by modifying substrate proteins. To identify USP13 substrates, we performed interactome analyses. We transfected human NCM460 and murine MODE‐K cells with Flag‐USP13 plasmids, followed by coimmunoprecipitation combined with LC‐MS/MS analysis to account for species differences in protein interactions (workflow shown in **Figure** **5**A). Notably, we identified 37 USP13‐interacting proteins in NCM460 cells (Dataset I, Figure 5B) and 135 proteins in MODE‐K cells (Dataset II, Figure 5C). By comparing these two interactomes, we identified five potential substrates of USP13, including GRP78, Ribosomal Protein L18 (Rpl18), Ribosomal Protein L18a (Rpl18a), Ribosomal Protein L23a (Rpl23a), and TOMM22 (Figure 5A–C). Among these candidates, the ER stress marker GRP78 (the protein corresponding to Hspa5) has been widely reported to be downregulated in human UC and DSS‐induced colitis mouse models.^[^ ^27^, ^28^ ^]^ Several compounds have been reported to exert protective effects in DSS‐induced colitis models by downregulating GRP78 expression, thereby reducing epithelial cell apoptosis and maintaining intestinal barrier function.^[^ ^29^, ^30^ ^]^ Therefore, we speculated that USP13 may target GRP78 when regulating UC.

Thus, we validated the interaction between endogenous USP13 and GRP78 in LPS‐treated NCM460 cells using co‐IP analysis (Figure 5D), as well as in Tm‐stimulated NCM460 cells (Figure 5E). Our data show that both LPS and Tm could induce the formation of the GRP78 and USP13 complex (Figure 5D,E). In addition, cellular co‐localization analysis showed significant colocalization of endogenous USP13 with GRP78 in LPS‐treated NCM460 cells (Figure 5F). Similarly, endogenous USP13 and GRP78 formed a complex in DSS‐induced colon tissues (Figure 5G). Also, the interaction between exogenous USP13 and GRP78 was confirmed in NIH/3T3 cells co‐transfected with Flag‐USP13 and His‐GRP78 plasmids (Figure 5H; Figure S4, Supporting Information) in a dose‐dependent manner (Figure 5I). Next, cellular co‐localization analysis showed significant colocalization of USP13 (green) with GRP78 (red) in NIH/3T3 cells (Figure 5J). These observations validated that USP13 interacts with GRP78 protein both in vitro and in vivo.

### 2.6 USP13 Deubiquitinates GRP78 at K327 via its Active Site C343, Modulating the Downstream ER Stress Pathway

**Figure 6:**
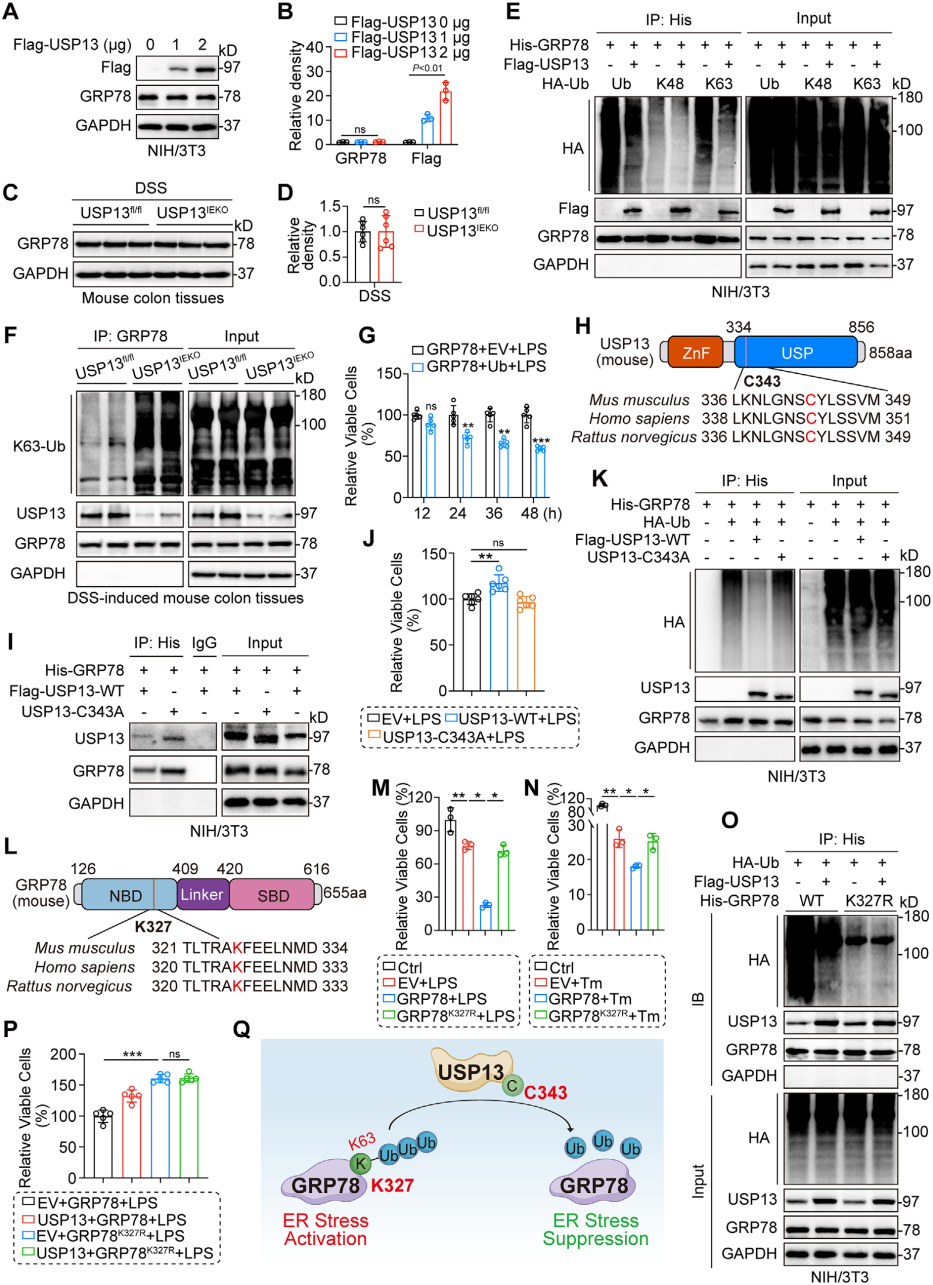
USP13 deubiquitinates GRP78 at K327 via its active site C343, modulating the downstream ER stress pathway. A,B) Representative blots A) and densitometric quantification B) of USP13 and GRP78 in NIH/3T3 cells transfected with Flag‐USP13 plasmids. C,D) Representative blots C) and densitometric quantification D) of GRP78 in colon tissues of mice with DSS‐induced colitis. E) Immunoprecipitation of GRP78 in NIH/3T3 cells co‐transfected with His‐GRP78, HA‐Ub, HA‐K48, and HA‐K63 plasmids. Ubiquitinated GRP78 was detected by immunoblotting with a GRP78‐specific antibody to clarify the ubiquitination pattern regulated by USP13. F) Immunoprecipitation of GRP78 in colon tissues in mice treated with DSS. G) CCK‐8 assay of viable MODE‐K cells overexpressing either EV or Ub plasmids, treated with 5 µg mL^−1^ LPS for different time periods (*n* = 5). H) Schematic illustration of USP13 active site (C343) mutation construct. I) Co‐IP of USP13 and GRP78 in NIH/3T3 expressing Flag‐USP13 (WT) or USP13 (C343A) and GRP78. J) CCK‐8 assay of viable MODE‐K cells overexpressing either EV, USP13 (WT), or USP13 (C343A) plasmids, treated with 5 µg mL^−1^ LPS for 24 h (*n* = 6). K) His‐GRP78 and HA‐Ub were transfected into NIH/3T3 with or without transfection of Flag‐USP13 (WT) or USP13 (C343A). Co‐IP was performed with anti‐His and followed by Western blot analysis of HA, USP13, and GRP78. L) Schematic illustration of the GRP78 ubiquitinated‐lysine residue (K327) mutation construct. M,N) CCK‐8 assay of viable MODE‐K cells overexpressing either EV, GRP78 (WT), or GRP78 (K327R) plasmids, treated with LPS (M, 5 µg mL^−1^) or Tm (N, 2.5 µg mL^−1^) for 24 h (*n* = 3). O) His‐GRP78 (WT or K327R) and HA‐Ub plasmids were transfected into NIH/3T3 with or without transfection of Flag‐USP13. Ubiquitinated GRP78 was enriched with anti‐His and detected with anti‐HA, anti‐USP13, and anti‐GRP78 antibodies. P) CCK‐8 assay of viable MODE‐K cells overexpressing either EV, USP13, or GRP78 (WT or K327R) plasmids, treated with LPS (5 µg mL^−1^) for 24 h (*n* = 5). Q) Scheme for the mechanism of USP13 deubiquitinates GRP78. Data are presented as mean ± SD; ^*^ *p* < 0.05; ^**^ *p* < 0.01; ^***^ *p* < 0.001; ns = not significant.

DUBs control cellular functions by altering the ubiquitination status of proteins, which influences protein stability and regulates signaling pathways.^[^ ^31^ ^]^ The interaction between USP13 and GRP78 raises the question of whether USP13 plays a regulatory role in GRP78. To answer this question, we first showed that GRP78 could be ubiquitinated (Figure S4A, Supporting Information), and USP13 could remove these ubiquitin tags (Figure S4B, Supporting Information). Interestingly, USP13 overexpression did not increase GRP78 protein levels (**Figure** **6**A,B) or affect GRP78 mRNA transcription (Figure S4C, Supporting Information). Additionally, USP13 did not stabilize GRP78 (Figure S4D,E, Supporting Information). Furthermore, we observed that USP13 deficiency in IECs did not affect the GRP78 expression in colon tissues of DSS‐treated mice (Figure 6C,D). Thus, we speculated that USP13 might influence the downstream signaling pathways of GRP78 rather than affecting its stability within cells. To identify the specific ubiquitin chains involved, we performed deubiquitination assays with various ubiquitin mutants. Our data show that USP13 specifically removed K63‐linked ubiquitin chains rather than the K48‐linked chains from GRP78 (Figure 6E). In vivo, USP13 deficiency in IECs during DSS treatment increased K63‐linked ubiquitination of GRP78 (Figure 6F). USP13 regulates multiple cellular processes through its deubiquitinase activity.^[^ ^32^, ^33^, ^34^ ^]^ Research shows that USP13 binds to IRHOM2 in liver cells and removes its K63‐linked ubiquitination, thus inhibiting its downstream activation cascade.^[^ ^32^ ^]^ This suggests that USP13 may protect against DSS‐induced colitis by modulating GRP78 signaling pathways. As expected, we found that the ubiquitinated state of GRP78 reduced the survival rate of NCM460 cells (Figure 6G). This suggested that the deubiquitinated state of GRP78 played a protective role in the LPS‐induced inflammation in colonic epithelial cells, consistent with our previous in vivo studies. The above data indicate that USP13 removes K63‐linked ubiquitin chains from GRP78, thereby reducing cell death in IECs.

The cysteine at site 343 (C343) is recognized as a crucial catalytic motif in the deubiquitinating function of USP13.^[^ ^35^ ^]^ The C343 residue, along with its surrounding residues, is conserved across multiple species, including *Mus musculus*, *Homo sapiens*, and *Rattus norvegicus* (Figure S4F, Supporting Information). To investigate the active site of USP13, we developed a mutant plasmid (USP13‐C343A) by substituting cysteine at site 343 with alanine in USP13 (Figure 6H). We observed that USP13‐C343A lost its protective effect on IECs under LPS stimulation compared to the USP13‐WT (Figure 6J). Moreover, USP13‐C343A showed diminished deubiquitinating ability on GRP78 (Figure 6K), although it still bound to GRP78 (Figure 6I). These findings highlight the critical role of C343 in USP13‐mediated deubiquitination of GRP78.

Our analysis elucidated the regulatory role of USP13 in deubiquitinating GRP78. To explore the specific deubiquitination site on GRP78 mediated by USP13, we analyzed the amino acid sequence of GRP78 using bioinformatics tools. Predictions from an online ubiquitination site prediction for GRP78 (Figure S4G, Supporting Information) and previous reports identified the conserved lysine 327 (K327) site in the nucleotide‐binding domain (NBD) of GRP78 as a potential ubiquitination site.^[^ ^36^ ^]^ Thus, we hypothesized that USP13 might predominantly target K327 for GRP78 deubiquitination. Thus, we constructed a K327R mutant (mutation of lysine to arginine at K327) of GRP78 (Figure 6L). Our data show that the K327R mutation did not affect the binding between USP13 and GRP78 (Figure S4H, Supporting Information). Compared to GRP78 (WT), GRP78 (K327R) restored the survival rate of IECs under LPS or Tm stimulation (Figure 6M,N), confirming the importance of the K327 site. However, the polyubiquitination level of GRP78 (K327R) was much lower than that of GRP78 (WT) and was not further reduced by USP13 (Figure 6O), suggesting that the lysine residue K327 of GRP78 is involved in USP13‐mediated GRP78 deubiquitination. GRP78 (K327R) increased cell survival under LPS treatment without needing further enhancement from USP13 (Figure 6P). In summary, our data reveal that USP13 reduced the K63‐linked polyubiquitination of GRP78 at position K327 via its active site C343 (Figure 6Q).

### 2.7 USP13 Ameliorates DSS‐Induced Colitis by Restoring the Intestinal Barrier and Suppressing Apoptosis Through Regulating GRP78

**Figure 7:**
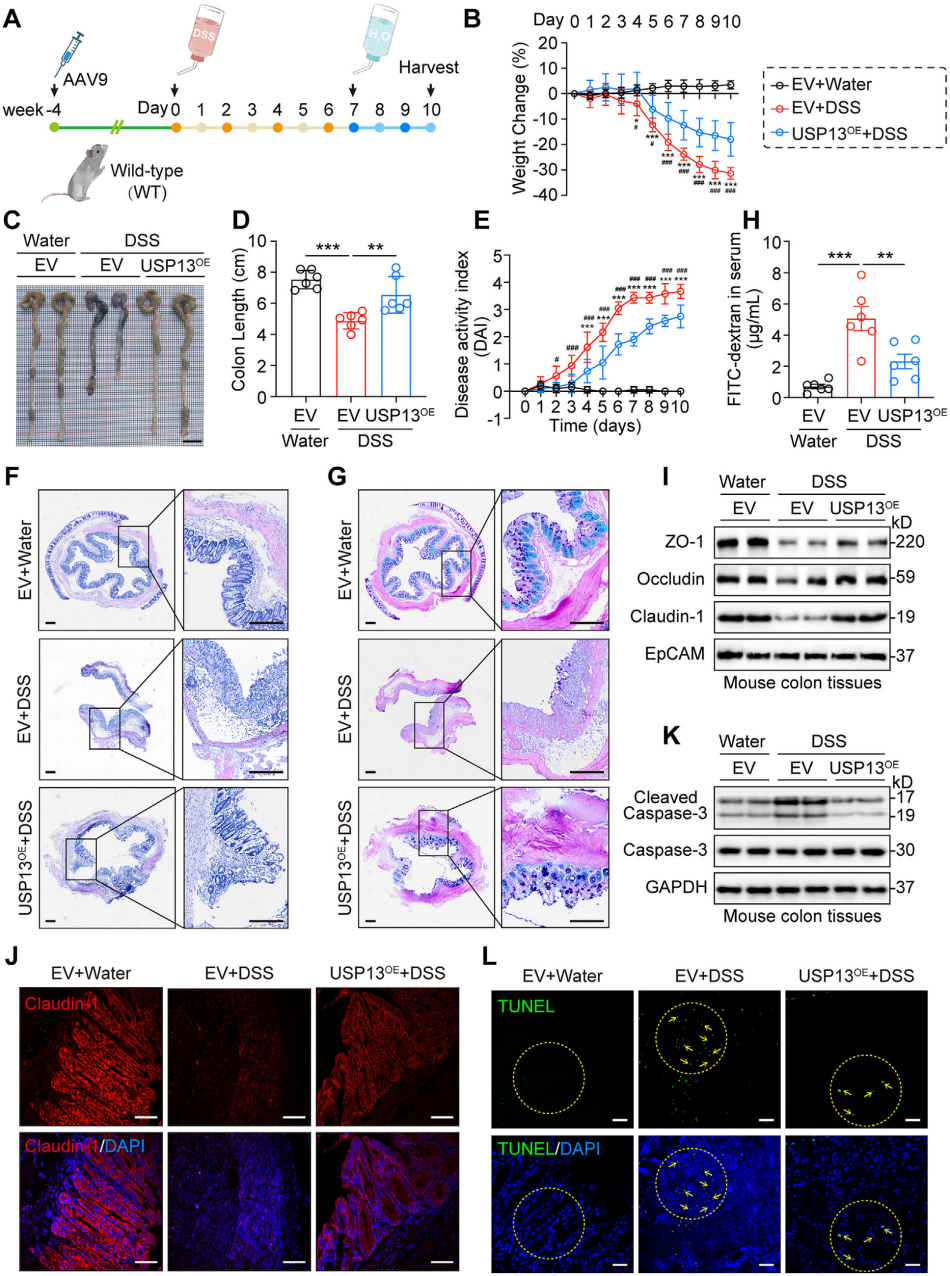
Over‐expression of USP13 alleviates colonic injury in the DSS‐induced colitis mouse model. A) AAV9 vectors carrying *Usp13* cDNA (USP13^OE^) were injected into the C57BL/6J mice via the tail vein, with the control group receiving an AAV9‐empty vector (EV). Four weeks postinjection, the colitis mouse model was initiated using 2.5% DSS. Body weight change B), gross morphology C), and colon length D) were determined on day 10 after DSS initiation (scale bar: 1 cm). E–G) Disease activity index E), representative H&E staining F), and AB‐PAS staining G) of colon tissues on day 10 (*n* = 6, scale bar: 200 µm). H) Intestinal permeability was assessed by FITC‐dextran assays on day 10 (*n* = 6). I) Representative blots of ZO‐1, Occludin, and Claudin‐1 proteins. EpCAM was used as the loading control. J) Representative immunofluorescence of Claudin‐1 (red) and DAPI (blue) in colon tissues with DSS‐induced colitis (scale bar: 100 µm). K) Representative blots of Cleaved Caspase‐3 and Caspase‐3 proteins. GAPDH was used as the loading control. L) Representative images of TUNEL staining in colon tissues on day 10 (scale bar: 100 µm). Data are presented as mean ± SD; for panels (B) and (E), ^*^ *p* < 0.05, ^***^ *p* < 0.001, EV+DSS versus EV group; ^#^ *p* < 0.05; ^###^ *p* < 0.001, EV+DSS versus USP13^OE^+DSS group; for panels (D) and (H), ^**^ *p* < 0.01, ^***^ *p* < 0.001.

Our previous data indicated a negative correlation between USP13 expression levels and the severity of DSS‐induced colitis, emphasizing the potential therapeutic role of USP13. To verify the function of USP13 in DSS‐induced colitis, WT mice were administered with AAV9 carrying *Usp13* cDNA (USP13^OE^) or AAV9‐empty vector (EV) for 4 weeks (**Figure** **7**A). The efficacy of AAV9 transduction was confirmed through immunoblotting (Figure S5A,B, Supporting Information). Notably, since AAV9 can induce long‐term transgene expression in the liver, we evaluated whether USP13^OE^ injection could cause toxicity in vivo, especially in the liver. The results indicated that USP13^OE^‐injected mice did not exhibit significant toxicity in the liver and other tissues compared to EV‐injected mice and untreated normal mice (Figure S5C, Supporting Information). As expected, compared to EV mice treated with DSS, USP13^OE^ mice exhibited reduced weight loss (Figure 7B), less colon shortening (Figure 7C,D), and lower DAI scores (Figure 7E). Histopathological examination and AB‐PAS staining confirmed the therapeutic effect of USP13 overexpression (Figure 7F,G; Figure S2B, Supporting Information). The FITC‐dextran assay, Western blot analysis of tight junction proteins, and Claudin‐1 immunofluorescence collectively demonstrated significant restoration of colonic mucosal barrier function (Figure 7H–J). Additionally, overexpression of USP13 significantly reduced DSS‐induced apoptosis in the colonic epithelium (Figure 7K,L). These findings emphasize the crucial role of USP13 in reducing susceptibility to DSS‐induced colitis by mitigating colonic injury, restoring the intestinal barrier, and suppressing apoptosis.

**Figure 8:**
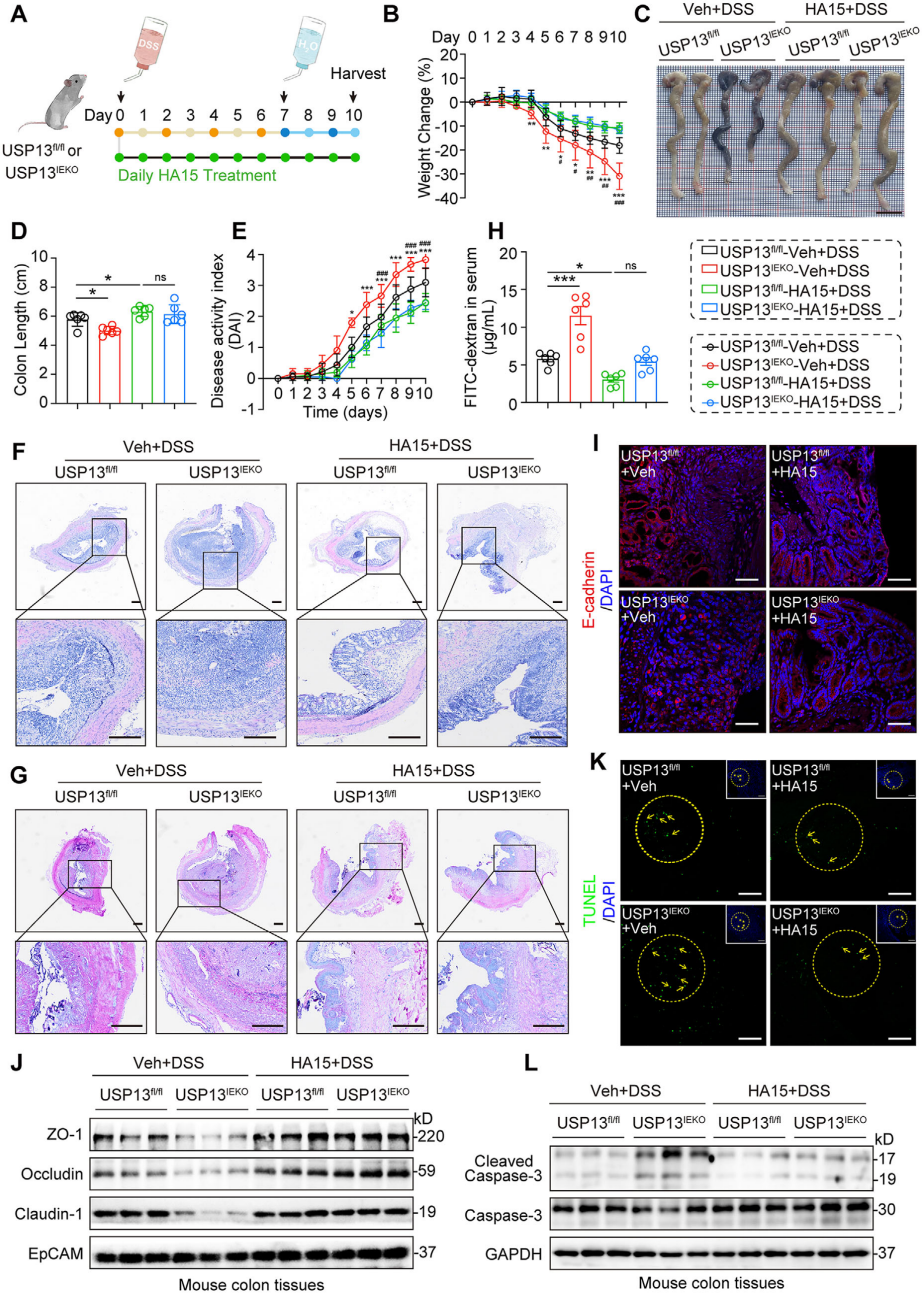
GRP78 inhibition suppresses DSS‐induced colonic ER stress, apoptosis, and injury regardless of the presence of USP13. A) USP13^IEKO^ or USP13^fl/fl^ mice received daily intraperitoneal injections of HA15 (10 mg kg^−1^) throughout the experiment, alongside 2.5% DSS treatment for 7 days, followed by 3 days of regular water. Body weight change B), gross morphology C), and colon length D) of colon tissues were determined on Day 10 after DSS initiation (scale bar: 1 cm). E) Disease activity index, representative H&E staining F), and AB‐PAS staining G) of colon tissues on day 10 (scale bar: 200 µm). H) Intestinal permeability was assessed by FITC‐dextran assays on day 10 (*n* = 6). I) Representative immunofluorescence of Claudin‐1 (red) and DAPI (blue) in colon tissues of DSS‐treated mice (scale bar: 100 µm). J) Representative blots of ZO‐1, Occludin, and Claudin‐1 proteins. EpCAM was used as the loading control. K) Representative TUNEL staining images in colon tissues with DSS‐induced colitis (scale bar: 100 µm). L) Representative blots of Cleaved Caspase‐3 and Caspase‐3 proteins. GAPDH was used as the loading control. Data are presented as mean ± SD; for panels (B) and (E), ^*^ *p* < 0.05, ^***^ *p* < 0.001, USP13^IEKO^‐Veh+DSS versus USP13^fl/fl^‐Veh+DSS group; ^#^ *p* < 0.05; ^##^ *p* < 0.01, ^###^ *p* < 0.001, USP13^fl/fl^‐Veh+DSS versus USP13^fl/fl^‐HA15+DSS group; for panels (D) and (H), ^*^ *p* < 0.05, ^***^ *p* < 0.001, ns = not significant.

Finally, we investigated the therapeutic effect of HA15, a specific GRP78 inhibitor, in the DSS‐induced colitis mouse model. As shown in **Figure** **8**A, USP13^IEKO^ and USP13^fl/fl^ mice received daily HA15 (10 mg kg^−1^) intraperitoneal injections throughout the experiment, alongside 2.5% DSS for 7 days, followed by 3 days of regular water. Our data show that HA15 significantly alleviated DSS‐induced colitis, evidenced by reduced weight loss (Figure 8B), less colon shortening (Figure 8C,D), and lower DAI scores (Figure 8E), regardless of the presence of USP13. Histopathological analysis and AB‐PAS staining showed that HA15 treatment significantly alleviated the DSS‐induced colonic injury (Figure 8F,G; Figure S2C, Supporting Information). Additionally, HA15 treatment reversed DSS‐induced increased intestinal permeability (Figure 8H), further confirmed by Claudin‐1 immunofluorescence (Figure 8I) and Western blot analysis of tight junction proteins (Figure 8J). Moreover, HA15 treatment significantly reduced DSS‐induced apoptosis (Figure 8K,L). These findings suggest that inhibition of GRP78 prevents USP13^IEKO^ mice from exacerbating DSS‐induced colonic injury, permeability disruption, and apoptosis, indicating that the therapeutic effect of USP13 depends on regulating the activation of GRP78. Thus, targeting USP13 or GRP78 could be a potential strategy for IBD treatment.

## 3 Discussion

Increasing evidence suggests that the dysregulation of E3 ubiquitin ligases and DUBs is closely associated with intestinal inflammation and colitis‐associated cancer due to their substrate specificity.^[^ ^31^ ^]^ In this study, we identified epithelial‐derived USP13 as a potential factor involved in UC pathogenesis. Immunoblotting showed USP13 downregulation in DSS‐induced colitis mice. GEO database analysis also revealed significantly reduced USP13 expression in inflamed colonic mucosa of UC patients. immunofluorescence co‐localization in both UC patients and mouse colon tissues further confirmed reduced USP13 expression in E‐cadherin⁺ intestinal epithelial cells compared to controls (Figure 1). Furthermore, the specific deletion of USP13 in IECs exacerbated DSS‐induced colonic injury, increased epithelial cell death, and disrupted the intestinal barrier in vivo. Mechanistically, we identified GRP78 as a crucial substrate of USP13 through interactome analysis in IECs. USP13 specifically reversed the K63‐linked ubiquitination of GRP78 at lysine 327 via its active site C343, thereby inhibiting ER stress activation and the resulting cellular apoptosis in IECs, thus protecting the intestinal barrier and preventing intestinal injury. Considering the specificity of USP13 in human and mouse colon tissues, we propose that therapies targeting intestinal epithelial cell USP13, such as AAVs carrying the *Usp13* gene, may offer new opportunities for treating UC.

Several DUBs contribute to the pathogenesis of IBD by altering the phenotype and function of intestinal cells. USP16 selectively removes K33‐linked polyubiquitin chains from nuclear factor kappa B kinase subunit beta (IKKβ), activating the NF‐κB signaling pathway.^[^ ^37^ ^]^ A20 promotes NF‐κB‐mediated cell death^[^ ^38^ ^]^ and enhances receptor‐interacting serine/threonine‐protein kinase 1‐dependent apoptosis in IECs,^[^ ^39^ ^]^ ultimately disrupting the intestinal barrier. Increased apoptosis of IECs in the lamina propria and reduced expression of tight junction proteins lead to greater intestinal barrier permeability during colitis.^[^ ^40^ ^]^ This allows more luminal antigens to cross the barrier and further activate innate immune cells in the gut, driving UC progression. In this study, mice with intestinal epithelial‐specific USP13 deficiency exacerbated colitis, evidenced by increased disruption of the intestinal epithelial barrier and elevated apoptosis of IECs. These data support the critical role of USP13 in IECs in UC.

Among the five potential substrates revealed by interactome analyses, Rpl18, Rpl18a, and Rpl23a are ribosomal proteins involved in mRNA translation and viral replication.^[^ ^41^, ^42^ ^]^ TOMM22 is a translocase of the outer mitochondrial membrane.^[^ ^43^ ^]^ As the role of these four proteins in IBD remains unclear, we did not focus on them for further investigation. The last potential candidate is GRP78, a chaperone heat shock protein regulating the UPR.^[^ ^44^ ^]^ Normally, GRP78 binds to and inactivates UPR pathway sensors (PERK, ATF6, and IRE1). However, ER stress from misfolded or unfolded proteins causes GRP78 to dissociate, activating the UPR. The uncontrolled ER stress can activate the downstream molecules, such as JNK and CHOP, leading to caspase‐dependent apoptosis. The role of ER stress‐mediated apoptosis in IBD has been documented.^[^ ^45^ ^]^ Studies have shown increased GRP78 expression in the colonic epithelium of animal models and patients with active IBD.^[^ ^4^, ^5^ ^]^ ER stress in IECs is a critical factor in intestinal inflammation. Up‐regulated ER stress in highly secretory IECs increases intestinal epithelial barrier permeability, promoting gut injury.^[^ ^46^ ^]^ These studies indicate that GRP78, as one of the key markers of ER stress, is upregulated during ER stress and plays a crucial role in ER stress‐mediated IEC apoptosis through the UPR pathways. Therefore, targeting GRP78 is considered a promising therapeutic strategy for IBD.

ER stress is crucially linked to cell apoptosis, inflammation, and tumor genesis. Recent studies focus on regulating ubiquitination levels of key proteins in the ER stress pathway, such as CHOP, PERK, IRE1, and ATF6. For example, tripartite motif‐containing protein 13 (TRIM13) reduces mesangial collagen synthesis in diabetic nephropathy through ubiquitination of CHOP.^[^ ^47^ ^]^ Similarly, the tumor‐suppressor breast cancer 1 (BRCA1) mediates proteasomal degradation by ubiquitinating PERK and IRE1.^[^ ^48^ ^]^ Moreover, ring finger protein 186 (RNF186) enhances the UPR by ubiquitinating ATF6.^[^ ^49^ ^]^ GRP78 exists in various forms, including free, bound, ubiquitinated, membrane‐bound, and secreted forms. Notably, GRP78, as an upstream regulator in the ER stress pathway, demonstrates superior regulatory potential through its ubiquitination levels. For example, regulating the ubiquitination levels of GRP78 has shown therapeutic potential in acute liver injury^[^ ^50^ ^]^ and cardiac fibrosis.^[^ ^51^ ^]^ Furthermore, GRP78 acts as a substrate for the deubiquitinase OTUD3^[^ ^52^ ^]^ and the ubiquitination target of TRIM21,^[^ ^53^ ^]^ playing a crucial role in tumor progression. These insights highlight the central role of GRP78 in regulating disease processes and its potential as a therapeutic target in disease management. In this study, we focused on ER and cytosolic GRP78 since deubiquitination mainly occurs there, while membrane‐bound GRP78 is minimally expressed in normal intestinal epithelial cells.^[^ ^54^ ^]^ While the critical role of ER stress‐mediated apoptosis in IBD has been widely reported, and increased GRP78 expression has been observed in the ileal and/or colonic epithelial tissues of animal models and patients with active IBD,^[^ ^4^, ^5^ ^]^ no study has elucidated the mechanism of targeting GRP78 for IBD treatment. Our study shows that USP13 inhibited ER stress‐mediated IEC apoptosis in a deubiquitination‐dependent manner. In vivo, USP13 deletion in IECs increased ER stress, UPR pathways, apoptosis, and colonic damage. LC‐MS/MS and co‐IP confirmed USP13 deubiquitinated GRP78, with C343 crucial for interaction. Additionally, we demonstrated that USP13 specifically reversed the K63‐linked ubiquitination of GRP78 at lysine 327 (Lys327), inhibiting ER stress and apoptosis.

In this study, we found that HA15 significantly alleviated DSS‐induced colitis, reduced epithelial apoptosis, and improved barrier function (Figure 8). Previous studies have demonstrated that HA15 selectively induces ER stress and apoptosis in tumor cells with minimal toxicity to normal cells due to their lower basal ER stress levels.^[^ ^55^, ^56^ ^]^ It has also been noted that the role of GRP78 and its downstream ER stress responses in colitis is influenced by context and stimulus intensity. Mild or adaptive ER stress helps restore epithelial homeostasis, while excessive ER stress may exacerbate inflammation.^[^ ^24^, ^57^, ^58^ ^]^ We speculate that HA15 exerts a protective effect by moderately inhibiting GRP78 activity, preventing the overactivation of ER stress. Consistently, our preliminary in vivo assessment (data not shown) confirmed that the dosage used was well tolerated without observable toxicity in major organs. Moreover, the protective effect of HA15 was abolished in USP13‐deficient mice, suggesting that USP13 may regulate GRP78 activity and cooperatively maintain ER homeostasis and epithelial integrity during colitis.

However, our study has some limitations. One of the limitations of this study is the inability to utilize an intestinal epithelial cell‐specific promoter for constructing the USP13‐carrying AAV. The large size of the USP13 gene precluded the effective use of the vil1 promoter, as it prevented normal construction. Even after removing the EGFP reporter gene, the packaging capacity of the vector was exceeded. Although the MUC2 promoter was effective, its large size also exceeded the packaging capacity. Given these constraints, it was recommended to employ a broad‐spectrum promoter to ensure the successful construction of the AAV. Nevertheless, we validated the overexpression efficiency of the AAV used in this study through USP13 immunoblotting in mouse colon tissues.

## 4 Conclusion

In summary, our study reveals that intestinal epithelial cell‐specific USP13 inhibits GRP78‐mediated ER stress activation and downstream apoptosis by specifically reversing K63‐linked ubiquitination of GRP78, thus protecting the intestinal barrier and preventing intestinal injury. We also demonstrated the protective role of the intestinal epithelial cell‐specific USP13‐GRP78 axis in IBD, suggesting that targeting USP13 for intestinal epithelial cell‐specific gene therapy may be a potential treatment strategy for IBD.

## 5 Experimental Section

### Reagents

Lipopolysaccharide (LPS, L4391) was purchased from Sigma‐Aldrich (St. Louis, MO). Bovine serum albumin (BSA, A8020) was purchased from Solarbio (Beijing, China) Tunicamycin (Tm, HY‐A0098) and cycloheximide (CHX, HY‐12320) were purchased from MedChemExpress (Monmouth Junction, NJ). Anti‐ HA (51064‐2‐AP), anti‐Flag (66008‐4‐Ig), anti‐6×His (His, 10001‐0‐AP), anti‐USP13 (16840‐1‐AP), anti‐GRP78 (11587‐1‐AP, 66574‐1‐lg), anti‐eukaryotic translation initiation factor 2‐alpha kinase 3 (PERK, 24390‐1‐AP), anti‐tight junction protein (ZO‐1, 21773‐1‐AP), anti‐Claudin‐1 (28674‐1‐AP), anti‐Occludin (27260‐1‐AP), anti‐DNA‐damage‐inducible transcript 3 (CHOP, 15204‐1‐AP), anti‐Cleaved Caspase‐3 (25128‐1‐AP), anti‐Caspase‐3 (19677‐1‐AP), anti‐activating transcription factor 4 (ATF4, 10835‐1‐AP), anti‐E‐cadherin (60335‐1‐Ig), and anti‐epithelial cell adhesion molecule (EpCAM, 21050‐1‐AP) antibodies were purchased from Proteintech (Wuhan, China). Anti‐Ki‐67 (Ki67, 9449T), anti‐endoplasmic reticulum to nucleus signaling 1 (IRE1α, 3294T), anti‐glyceraldehyde‐3‐phosphate dehydrogenase 1 (GAPDH, 5174S) antibodies, α‐Tubulin (2144S) were purchased from Cell Signaling Technology (Danvers, MA). Anti‐USP13 (ab109264), anti‐activating transcription factor 6 (ATF6, ab122897), Goat Anti‐Rabbit IgG H&L (Alexa Fluor 647) (ab150079) and Goat Anti‐Mouse IgG H&L (Alexa Fluor 647) (ab150115), Goat Anti‐Rabbit IgG H&L (Alexa Fluor 488) (ab150077) and Goat Anti‐Mouse IgG H&L (Alexa Fluor 488) (ab150113) were purchased from Abcam (Cambridge, UK). Protein A+G Agarose (P2012), HRP‐linked antirabbit secondary antibody (A0208), and antimouse secondary antibody (A0216) were purchased from Beyotime (Shanghai, China).

### Animal Experiments

All animal care and experimental procedures were approved by the Animal Ethics and Welfare Committee of Hangzhou Normal University (Hangzhou, China; approval number: HSD20230302). All mice were bred and maintained in a specific pathogen‐free room, and all experiments were performed using 6‐ to 8‐week‐old male mice on C57BL/6J background. The intestinal epithelial‐specific *Usp13* knockout (USP13^IEKO^) mice were generated by GemPharmatech Co., Ltd (Nanjing, China). The genotype of USP13^IEKO^ was maintained by crossing the C57BL/6JGpt‐ *Usp13^em1Cflox^*/Gpt mouse (*Usp13^fl/fl^*, strain No. T005058) and the C57BL/6JGpt‐*H11^em1Cin(Vil1‐icre)^*/Gpt mouse (*Vil1‐iCre*, strain No. T004714). Cre‐negative littermates were used as wild‐type (WT) controls. All mice were acclimatized to the laboratory for one week before initiating the studies.

1. For the DSS‐induced mouse colitis model, 6‐ to 8‐week‐old WT or USP13^IEKO^ male mice were treated with 2.5% (weight/volume) DSS (36000 to 50000 MW; 021 601 1080, MP Biomedicals, Irvine, CA) in drinking water for 7 days, followed by 3 days of regular water. Animals exhibiting health concerns unrelated to the study conditions were excluded from the analysis. Body weight was monitored daily, and rectal bleeding and stool consistency were measured and scored as previously reported.^[^ ^10^ ^]^ Disease activity index (DAI) scores were determined based on body weight loss, occult blood, and stool consistency, with each parameter scored from 1 to 4 (Table S1, Supporting Information).^[^ ^59^ ^]^ Gross photos were collected using a camera. Blood samples and dissected colon tissues were collected for subsequent analysis. The survival rate was recorded.
2. The *Usp13* overexpression system was established using AAV9. The AAV9 carrying *Usp13* cDNA (NM_001013024.2) alongside a promoter (CMV‐betaGlobin‐MCS‐3Flag‐SV40 Poly A, GV411) was constructed by Genechem (Shanghai, China). The AAV9 carrying *Usp13c* DNA (USP13^OE^, 2 × 10^11^ vg) was injected into 4‐week‐old wild‐type male mice via the tail vein, while the control group received AAV9‐empty vector (EV, 2 × 10^11^ vg). Four weeks postinjection of USP13^OE^ and EV,^[^ ^60^, ^61^ ^]^ DSS‐induced mouse colitis modeling was initiated with 2.5% DSS treatment for 7 days, followed by 3 days of regular water. The tissue samples were collected as described in (1).
3. For the GRP78 inhibitor‐treated DSS‐induced mouse colitis model, HA15 (H873084, Shanghai Macklin Biochemical, Shanghai, China), a specific GRP78 inhibitor, was used to confirm the therapeutic effect of GRP78 blockade in the DSS‐induced mouse colitis model. HA15 (10 mg kg^−1^) was dissolved in 2.5% dimethyl sulfoxide (DMSO) solution (diluted in saline) and intraperitoneally injected at 10 µL g^−1^ body weight. The control group received the same volume of 2.5% DMSO solution (diluted in saline). The HA15‐treated group received daily intraperitoneal injections of HA15 (10 mg kg^−1^) until the end of the experiment, with 2.5% DSS treatment for 7 days, followed by 3 days of regular water.

### Intestinal Permeability Assay

Before the experiment, DSS‐treated mice were deprived of food and water for 4 h. FITC‐dextran (MW 4000, HY‐128868A, MedChemExpress) was prepared at a dose of 0.6 mg g^−1^ body weight, dissolved in 200 µL phosphate‐buffered saline (PBS), and administered via oral gavage. Four hours post‐gavage, mice were euthanized, and blood samples were collected. After being maintained at room temperature for 1 h, the serum was separated by centrifugation. The fluorescence intensity was measured using a SpectraMax plate reader (Silicon Valley, San Jose, CA) within 4 h, with excitation at 488 nm and emission at 525 nm. Intestinal permeability was measured by the amount of FITC‐dextran translocated from the intestine to the bloodstream.

### Cell Culture and Transfection

Human normal colon epithelial cell line NCM460 (BNCC339288) and murine intestinal epithelial cell line MODE‐K (BNCC338300) were purchased from the BeNa Culture Collection (Beijing, China). The murine cell line, NIH/3T3 (GNM 6), was purchased from the National Collection of Authenticated Cell Cultures (Shanghai, China). MODE‐K and NIH/3T3 cells were cultured in Dulbecco's modified Eagle's medium (DMEM, C11995500BT, Gibco, Waltham, MA) supplemented with 10% fetal bovine serum (FBS, 10099–141, Gibco) and 1% penicillin/streptomycin (BC‐CE‐007, Biochannel, Nanjing, China). NCM460 cells were cultured in RPMI 1640 (C11875500BT, Gibco) supplemented with 10% FBS and 1% penicillin/streptomycin.

Expression plasmids for Flag‐USP13 (human or mouse), USP13‐C343A (mouse), His‐GRP78 (mouse), His‐GRP78‐K327R (mouse), HA‐Ub (mouse), HA‐K63 (mouse), and HA‐K48 (mouse) were constructed by Genechem (Shanghai, China). Transfection of expression plasmids was performed using LipofectAMINE 3000 (L3000150, Thermo Fisher Scientific, Cleveland, OH).

### Coimmunoprecipitation (co‐IP) Combined with LC‐MS/MS Analysis (Interactome)

NCM460 or MODE‐K cells were transfected with Flag‐empty vector (EV) or Flag‐USP13 (USP13^OE^) plasmids, followed by stimulation with LPS (5 µg mL^−1^). Anti‐Flag and protein G‐Sepharose beads were used for co‐IP from cell lysates. Binding proteins were extracted from the co‐IP beads using SDT lysis buffer and then digested into peptides using the FASP method. LC‐MS/MS analysis was performed by BIOPROFILE (Shanghai, China), and potential substrate proteins were identified based on the intensity of detected proteins.

### Protein Co‐Localization

For co‐localization of USP13 and GRP78 in vitro, LPS‐stimulated NCM460 cells or NIH/3T3 cells transfected with expression plasmids were incubated with anti‐USP13 (1:200, 16840‐1‐AP) and anti‐GRP78 (1:200, 66574‐1‐lg) antibodies overnight at 4 °C. Next, cells were incubated with Alexa Fluor647‐labeled secondary antibody and Alexa Fluor488‐labeled secondary antibody for 1 h at room temperature. Nuclei were stained with DAPI, and images were captured using laser confocal microscopy (C2si, Nikon, Tokyo, Japan).

### Transcriptome Sequencing

Samples for transcriptome analysis were collected from the colon tissues of DSS‐induced WT mice and control WT mice. Total RNA was extracted from colon tissues using TRIzol reagent (Thermo Fisher Scientific). Library construction, sequencing, and GSEA enrichment analysis were conducted by LC‐Bio Technologies Co., Ltd (Hangzhou, China).

### Cell Viability Assay

Cells were seeded at a density of 5 × 10^3^ per well in 96‐well plates. After seeding, cells were transfected with either EV or USP13^OE^ plasmids. Following transfection, cells were treated with LPS at a concentration of 2.5 µg mL^−1^ for various periods (e.g., 12, 24, 36, and 48 h). Alternatively, cells were transfected with either EV or USP13^OE^ plasmids and then treated with different concentrations of LPS (e.g., 1.25, 2.5, 5.0, 10 µg mL^−1^) for 48 h. Untreated cells served as controls. Cell viability was assessed using the CCK‐8 assay (PF00004, Proteintech) according to the manufacturer's instructions. Briefly, CCK‐8 reagent was added to each well and incubated for 2 h at 37 °C. Absorbance was measured at 450 nm using a SpectraMax plate reader.

### Immunofluorescence

Cells were seeded at a density of 5 × 10^3^ cells per well in confocal culture dishes (NS801001‐1, NEST, Wuxi, China). Following seeding, cells were either transfected with EV, USP13^OE^ plasmids, or vectors expressing NC and siUSP13. After transfection, cells were treated with 1 µg mL^−1^ LPS for 48 h. Cells were then fixed with 4% paraformaldehyde for 10 min and permeabilized with 0.1% Triton X‐100 for 10 min. Next, cells were incubated overnight at 4 °C with anti‐E‐cadherin and anti‐ZO‐1 antibodies (diluted 1:200 in 1% BSA). Subsequently, cells were incubated with Alexa Fluor647‐labeled secondary antibody and Alexa Fluor488‐labeled secondary antibody for 1 h at room temperature. Samples were mounted using an antifade mounting medium containing DAPI for nuclear staining. Images were captured and analyzed using a Zeiss LSM 900 laser confocal microscope (Zeiss, Oberkochen, Germany) and Zeiss ZEN software.

### Human Colon Tissue Collection

Colon biopsy samples were collected from 11 patients diagnosed with UC and 6 healthy individuals undergoing colonoscopic examination. The diagnosis of UC was confirmed based on both endoscopic and histopathological findings, and only patients with moderate to severe active disease were included. This study was approved by the Affiliated Xiangshan Hospital of Wenzhou Medical University (Approval number: XYYJ‐2024‐645). Detailed clinical information, including age, sex, disease severity, diagnostic basis, and treatment history, is summarized in Table S2 (Supporting Information).

### Histological Analysis

Colon tissues were fixed in 4% paraformaldehyde, embedded in paraffin, and sectioned at a thickness of 5 µm. The sections were stained with hematoxylin and eosin (H&E, G1120, Solarbio) according to the manufacturer's instructions. Additional staining was performed using Alcian Blue/Phosphoric Acid Schiff (AB/PAS, G1285, Solarbio). Images were captured using a bright‐field microscope (TE2000, Nikon). Histological injury of the colon was assessed using a standardized H&E‐based scoring system, including four parameters: epithelium loss, crypt damage, goblet cell depletion, and inflammatory cell infiltration. Each parameter was scored from 0 to 3, with a total score ranging from 0 to 12 (Table S4, Supporting Information).

Immunoreactivity was detected using 3,3′‐diaminobenzidine (DAB). The colon sections underwent deparaffinization and rehydration, followed by blocking with 5% BSA. They were then incubated overnight at 4 °C with primary antibodies, including anti‐USP13 (1:100 dilution with 1% BSA), anti‐ATF4 (1:200 dilution with 1% BSA), anti‐CHOP (1:100 dilution with 1% BSA), and anti‐Cleaved Caspase‐3 (1:200 dilution with 1% BSA). After primary antibody incubation, sections were treated with horseradish peroxidase‐labeled secondary antibody for 2 h at room temperature, followed by DAB staining for 2 min at room temperature. Images were captured using a bright‐field microscope (80i, Nikon). Quantification of staining intensity was performed using the IHC Profiler plugin in ImageJ. The percentage of cells with high positive, positive, low positive, and negative staining was obtained, and an H‐score was calculated using the following formula: H‐score = (% of low positive × 1) + (% of positive × 2) + (% of high positive × 3), as previously described.^[^ ^62^ ^]^

### TUNEL Analysis

Apoptosis in colon tissue sections was evaluated using the TUNEL apoptosis detection kit (E‐CK‐A321, Elabscience, Wuhan, China) according to the manufacturer's instructions.

### Western Blot and Co‐Immunoprecipitation (co‐IP)

Total protein was extracted from cells or colon tissue lysates, separated on 10% sodium dodecyl sulfate (SDS)‐polyacrylamide gels, and then electro‐transfer onto polyvinylidene fluoride (PVDF) membranes. The membranes were blocked with skim milk for 1 h at room temperature, followed by incubation with primary antibodies overnight at 4 °C. Secondary antibodies were then applied for 2 h at room temperature.

For co‐IP, cells or tissue lysates were incubated with primary antibody overnight at 4 °C, with a portion of the lysate retained as an input sample. The protein lysates were precipitated using protein A + G agarose beads (P2012, Beyotime). Control rabbit IgG (ab172730, Abcam), Control mouse IgG (ab190475, Abcam), HRP‐labeled anti‐mouse (ab131368, Abcam) and anti‐rabbit (ab131366, Abcam) secondary antibodies were applied for 2 h at room temperature. Interacting proteins were detected by immunoblotting.

### Real‐Time Reverse Transcriptase‐Polymerase Chain Reaction (RT‐qPCR) Assay

Total RNA was extracted from cells and lung tissues using Trizol (Invitrogen, Carlsbad, CA). The reverse transcription of isolated RNA was tested using PrimeScript RT reagent Kit (TaKaRa, Tokyo, Japan). RT‐qPCR was carried out using the Eppendorf Realplex 4 instruments (Eppendorf, Hamburg, Germany). Primers were synthesized and obtained from Sangon Biotech (Shanghai, China). The primer sequences for all genes are shown in Table S3 (Supporting Information). The relative mRNA levels of each gene were normalized to the levels of *Actb*.

### Gene Silencing Using siRNA

Gene expression was silenced using the siRNA technique. *Usp13* siRNA was designed by RiboBio (Guangzhou, China). Transfection of NCM460 cells with siRNA was performed using LipofectAMINE 2000 (11 668 500, Thermo Fisher Scientific) according to the manufacturer's instructions. The target sequence for si‐h‐USP13 was 5′‐CCAAGCACTTAGCGCATTT‐3′.

### RNA Sequencing Data From the GEO Database

Transcriptomic data from patients with UC were obtained from the NCBI Gene Expression Omnibus (GEO) database (https://www.ncbi.nlm.nih.gov/geo/). Two datasets were analyzed in this study: GSE36807 (Platform: GPL570; sample IDs: GSM901319‐GSM901338) and GSE48634 (Platform: GPL10558; sample IDs: GSM1182603‐GSM1182736), to compare USP13 RNA expression levels in colonic tissues between UC patients and healthy controls.^[^ ^63^, ^64^ ^]^

### Statistical Analysis

Data were presented as mean ± SD. Image quantification was performed using ImageJ software (NIH, Bethesda, MD). Statistical analysis was performed using GraphPad Prism 7.0 software (version 7.0, San Diego, CA). A two‐tailed Student's *t*‐test was used to compare the differences between the two groups. For comparisons among multiple groups, one‐way ANOVA followed by Dunnett's multiple comparisons test was applied. All statistical details regarding *p*‐value and *n* can be found in the figure legends and expanded figure legends. No animals were excluded from the study.

## Conflict of Interest

The authors declare no conflict of interest.

## Author Contributions

C.Q., Z.W., and Y.W. were responsible for the concept and experimental design. C.Q., C.H., Y.X., W.X., WT, and A.S. conducted the experiments. L.H., W.G., and C.Q. contributed to clinical sample collection. C.Q., C.H., Y.X., and W.X. helped with the animal study. L.H., Z.W., W.G., W.T., and A.S. performed statistical analysis. C.Q. and Y.W. wrote and edited the manuscript. Y.W. is the guarantor for this paper. All authors read and approved the final manuscript.

## Supporting information



Supporting Information

## Data Availability

The data that support the findings of this study are available from the corresponding author upon reasonable request.
