# Supplementary material for: Intestinal Epithelial‐Derived USP13 Alleviates Colonic Inflammation by Suppressing GRP78‐mediated Endoplasmic Reticulum Stress
Source: Adv Sci (Weinh). 2025 Jul 18;12(38):e00741. doi: 10.1002/advs.202500741 (PMC12520541; doi:10.1002/advs.202500741)
Supplement: Supplementary file 1 — Supporting Information [file ADVS-12-e00741-s001.docx]

**Supporting Information for**

**Intestinal epithelial-derived USP13 alleviates colonic inflammation by suppressing GRP78-mediated endoplasmic reticulum stress**

**The supplementary file contents**

Supplementary tables (S1-S4)

Supplementary figures (S1-S5)**1. Supporting tables**

**Table S1.** Disease Activity Index (DAI) Evaluation System

| **DAI** | **% Weight loss** | **Stool consistency** | **Rectal bleeding** |
| --- | --- | --- | --- |
| **0** | None | Normal | None |
| **1** | 1-5% |  | Occult blood ± |
| **2** | 6-10% | Loose stool | Occult blood + |
| **3** | 11-20% |  | Occult blood ++ |
| **4** | ＞20% | Diarrhea | Gross blood |

**Table S2.** Clinical and Endoscopic Characteristics of Enrolled Ulcerative Colitis Patients

| **Patient**  **ID** | **Sex/Age** | **UC Type** | **Clinical**  **Type** | **Disease**  **Severity** | **Disease**  **Stage** | **Endoscopic Findings** | **Pathological**  **Features** | **Diagnostic**  **Basis** | **Inclusion**  **Criteria Status** | **Exclusion**  **Criteria Status** |
| --- | --- | --- | --- | --- | --- | --- | --- | --- | --- | --- |
| 21-41598 | Male /  72 y | Pancolitis | Chronic  relapsing | Moderate to  Severe | Active phase | Inflammatory changes in entire colonic mucosa | Chronic inflammation with crypt abscesses and exudate | Endoscopy + Pathology | Active phase, pathology consistent with UC | No abdominal pain or fever |
| 21-2328 | Female /  39 y | Pancolitis | Acute  onset | Moderate | Active phase | Extensive mucosal inflammatory ulceration of the colon | Chronic inflammation with crypt abscesses and granulation tissue | Endoscopy + Pathology | Active phase, consistent with UC pathology | No specific exclusion criteria |
| 24-20459 | Female /  51 y | Pancolitis | Chronic  relapsing | Severe | Active phase | Pancolonic congestion, edema, ulceration, and pseudopolyps | Heavy lymphoplasmacytic infiltration, EBV(+) | Endoscopy + Pathology + EBER | Diagnosed UC, active phase, severe | No obvious exclusion criteria |
| 24-3671 | Male /  27 y | Pancolitis | Chronic  relapsing | Moderate | Active phase | Diffuse erosion of colonic mucosa | Plasma cells, eosinophils, crypt abscesses, partial glandular distortion | Endoscopy + Pathology | Meets clinical features of active UC | No specific exclusion criteria |
| 24-04852 | Male /  Unknown | Pancolitis with terminal ileum involvement | Chronic  relapsing | Moderate | Active phase | Patchy erosion in colon and terminal ileum ulcer | Neutrophilic infiltration and crypt abscess formation | Endoscopy + Pathology | Diagnosed UC, active phase | No obvious exclusion criteria |
| 24-17776 | Male /  24 y | Left-sided colitis | Acute  onset | Moderate | Active phase | Erosion in sigmoid colon and rectum | Cryptitis, necrosis, and inflammatory cell infiltration | Endoscopy + Pathology + Mayo score | Diagnosed UC, active phase, Mayo score moderate | No infection or immunodeficiency |
| **Patient**  **ID** | **Sex/Age** | **UC Type** | **Clinical**  **Type** | **Disease**  **Severity** | **Disease Stage** | **Endoscopic Findings** | **Pathological**  **Features** | **Diagnostic**  **Basis** | **Inclusion**  **Criteria Status** | **Exclusion**  **Criteria Status** |
| 22-31996 | Male /  75 y | Pancolitis | Chronic  relapsing | Moderate | Active phase | Multiple superficial mucosal ulcers in the colon and rectum | Chronic inflammation with neutrophilic infiltration and crypt abscesses | Endoscopy + Pathology | Diagnosed UC, active phase | No abdominal distension or fever |
| 23-34176 | Female /  Unspecified | Pancolitis | Acute  onset | Moderate | Active phase | Diffuse congestion and erosion in the entire colon | Neutrophilic infiltration, crypt abscesses, and granulation tissue | Endoscopy + Pathology | Diagnosed UC, active phase, pathological features consistent | No specific exclusion criteria |
| 21-18032 | Female /  57 y | Pancolitis | Chronic  relapsing | Mild to  Moderate | Active phase | Diffuse pancolonic mucosal hyperemia and erosion | Chronic inflammation with crypt abscess formation | Endoscopy + Pathology | Meets UC pathological features, active phase | No fever or hematochezia |
| 24-04211 | Female /  42 y | Pancolitis | Chronic  relapsing | Moderate | Active phase | Rectal mucosal congestion and erosion with loss of vascular pattern, colon severely distorted; follow-up showed pseudopolyps | Abundant neutrophils and crypt abscess formation | Endoscopy + Pathology | Diagnosed UC, active phase, moderate | No infection, tumor, or immunodeficiency |
| 24-11054 | Female /  67 y | Distal colitis | Chronic  relapsing | Moderate | Active phase | Inflammatory changes in rectum and sigmoid colon | Heavy infiltration of neutrophils and plasma cells | Endoscopy + Pathology | Diagnosed UC, active phase | No obvious exclusion criteria |

**Table S3.** Sequences of primers used for real-time qPCR assay

| **Gene** | **Forward** | **Reverse** |
| --- | --- | --- |
| *Tjp1* (mouse) | ACCCGAAACTGATGCTGTGGATAG | AAATGGCCGGGCAGAACTTGTGTA |
| *Ocln* (mouse) | GGACCCTGACCACTATGAAACAGACTAC | ATAGGTGGATATTCCCTGACCCAGTC |
| *cldn1* (mouse) | CCCTTCAGCAGAGCAAGGTT | TAGGGCAACCAAGTGCCTTT |
| *Usp13* (mouse) | TCCCAGAGTTCCAGAGAGCGTATG | TGTGTGTTGAAGTCCTGCGTTGG |
| *Usp18* (mouse) | GACCAGATCACGGACACAGACTTG G | GTTTGCTCCTCCTGCTGCTCTC |
| *Otud1* (mouse) | TCTGCCTGGCTGCTGGAAGAG | GTGCTCGCTCAGTCGGAAGTTC |
| *Otulinl* (mouse) | GATGTCAACCGCTTCACCCTGAG | CTGTCTCCTTGGCATGTTCCCTTGC |
| *Uchl4* (mouse) | ATGCCTGTGGAACGATTGGAACG G | TGGCTCTCTCTTCAGGGCTCATTG |
| *Hspa5* (mouse) | CCGAGGAGGAGGACAAGAAGGAG | GAACACACCGACGCAGGAATAGG |
| *Actb* (mouse) | CTACCTCATGAAGATCCTGACC | CACAGCTTCTCTTTGATGTCAC |
| *DNAJB9* (human) | AGGACAAAGAGGTAGTGGAAGTTC | CATCCTGGCGTGTCTGGAAATG |
| *ATF4* (human) | GTCTGCCCGTCCCAAACCTTAC | TCCTGCTCCGCCCTCTTCTTC |
| *Xbp1s* (mouse/human) | GGTCTGCTGAGTCCGCAGCAGG | GAAAGGGAGGCTGGTAAGGAAC |
| *USP13* (human) | AGCGGAGGCAGGAAGATGGC | ATTGGGAGAGTCGTAGGAGAAGGC |
| *HSPA5* (human) | CCGAGGAGGAGGACAAGAAGGAG | ACACGCCGACGCAGGAGTAG |
| *ACTB* (human) | CCTGGCACCCAGCACAAT | GCCGATCCACACGGAGTACT |

**Table S4.** Histological scores of dextran sulfate sodium (DSS)-induced colitis.

| **Histological features** | **Score** | **Description** |
| --- | --- | --- |
| **Epithelium loss** | 0 | None |
|  | 1 | 0.0-5.0% |
|  | 2 | 5.0-10.0% |
|  | 3 | ＞10.0% |
| **Crypt damage** | 0 | None |
|  | 1 | 0.0-10.0% |
|  | 2 | 10.0-20.0% |
|  | 3 | ＞20.0% |
| **Depletion of goblet cells** | 0 | None |
|  | 1 | Mild |
|  | 2 | Moderate |
|  | 3 | Severe |
| **Infiltration of inflammatory cells** | 0 | None |
|  | 1 | Mild |
|  | 2 | Moderate |
|  | 3 | Severe |

1. Supporting figures

**
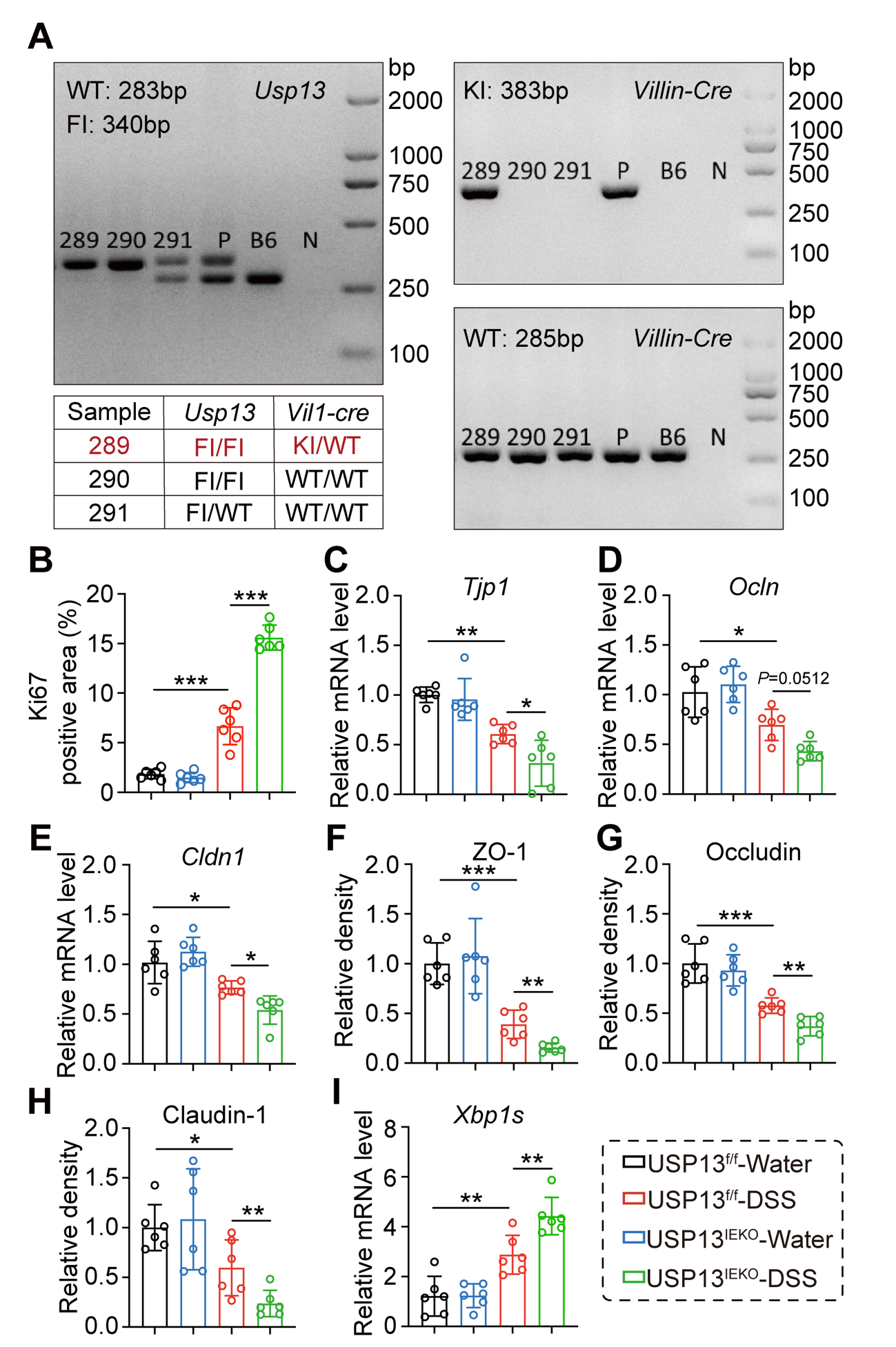
**

**Figure S1. Intestinal epithelial-specific USP13 deficiency aggravates DSS-induced colitis *in vivo*. (A)** The primers of *Usp13*^fl/fl^ (WT: 283 bp, FI: 340 bp) and Villin-Cre (WT: 285 bp, KI: 383 bp) were respectively used for PCR to identify the genotype of mice. (P: Positive control; B6: Negative control; N: No-template control). **(B)** Quantification of Ki67 (n = 6). **(C-E)** The mRNA levels of *Tjp1*, *Ocln*, and *Cldn1* were measured via RT-qPCR assay. Data were normalized to the levels of *Actb* (n = 6). **(F-H)** Densitometric quantifications for Fig. 2K (n = 6). **(I)** The mRNA levels of *Xbp1s* were measured via RT-qPCR assay. Data were normalized to the levels of *Actb* (n = 6). Data are presented as mean ± SD. Statistical significance was determined by One-way ANOVA followed by Dunnett's multiple comparisons test. * *P* < 0.05; ** *P* < 0.01; ns = not significant.

**
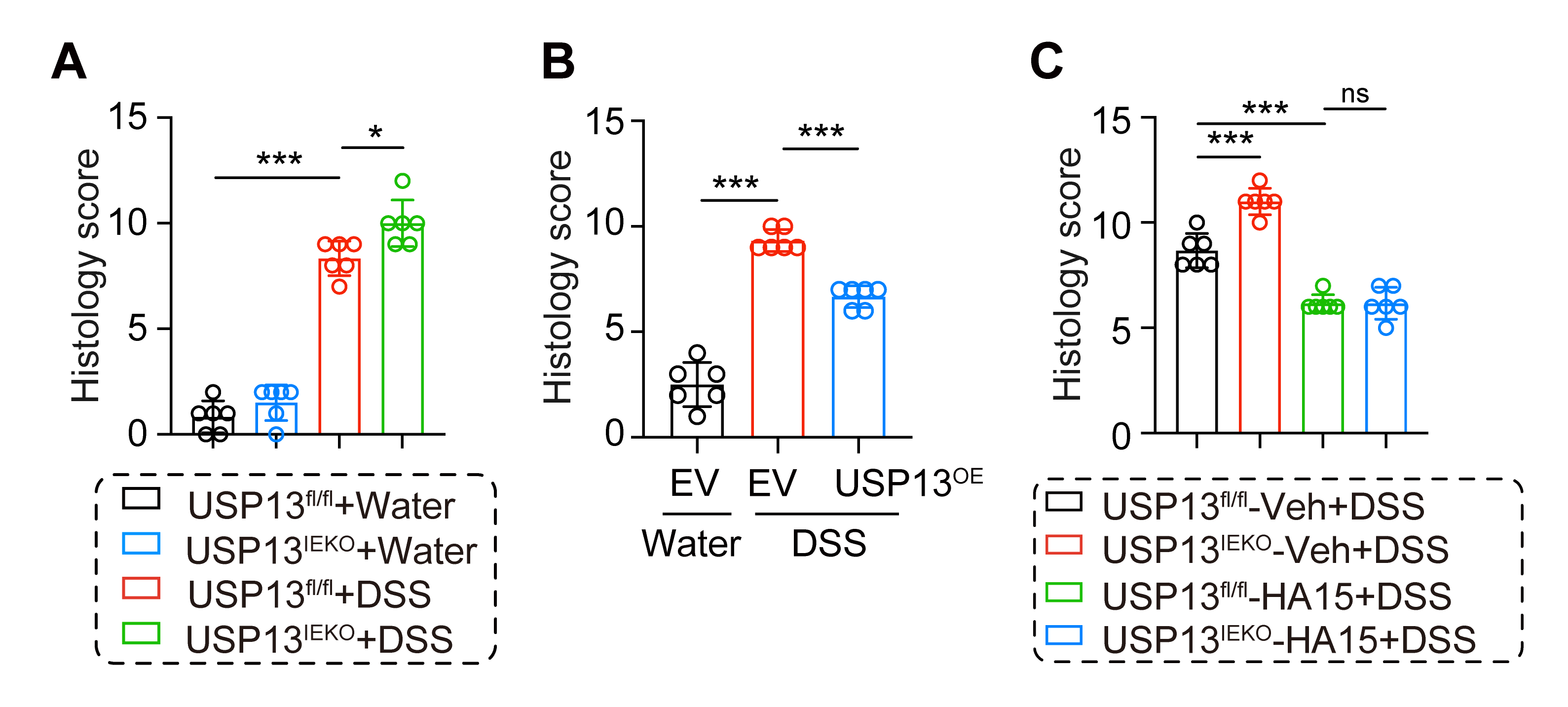
**

**Figure S2*.* Histological scores of colon tissues from different experimental groups. (A)** Histology scores for USP13^fl/fl^ + Water, USP13^IEKO^+Water, USP13^fl/fl^ + DSS, and USP13^IEKO^+DSS. **(B)** Histology scores for EV + Water, EV + DSS, and USP13^OE^ + DSS. **(C)** Histology scores for USP13^fl/fl^-Veh + DSS, USP13^IEKO^-Veh + DSS, USP13^fl/fl^-HA15 + DSS, and USP13^IEKO^-HA15 + DSS. Data are presented as mean ± SD. Statistical significance was determined by One-way ANOVA followed by Dunnett's multiple comparisons test. * *P* < 0.05; *** *P* < 0.001; ns = not significant.

**
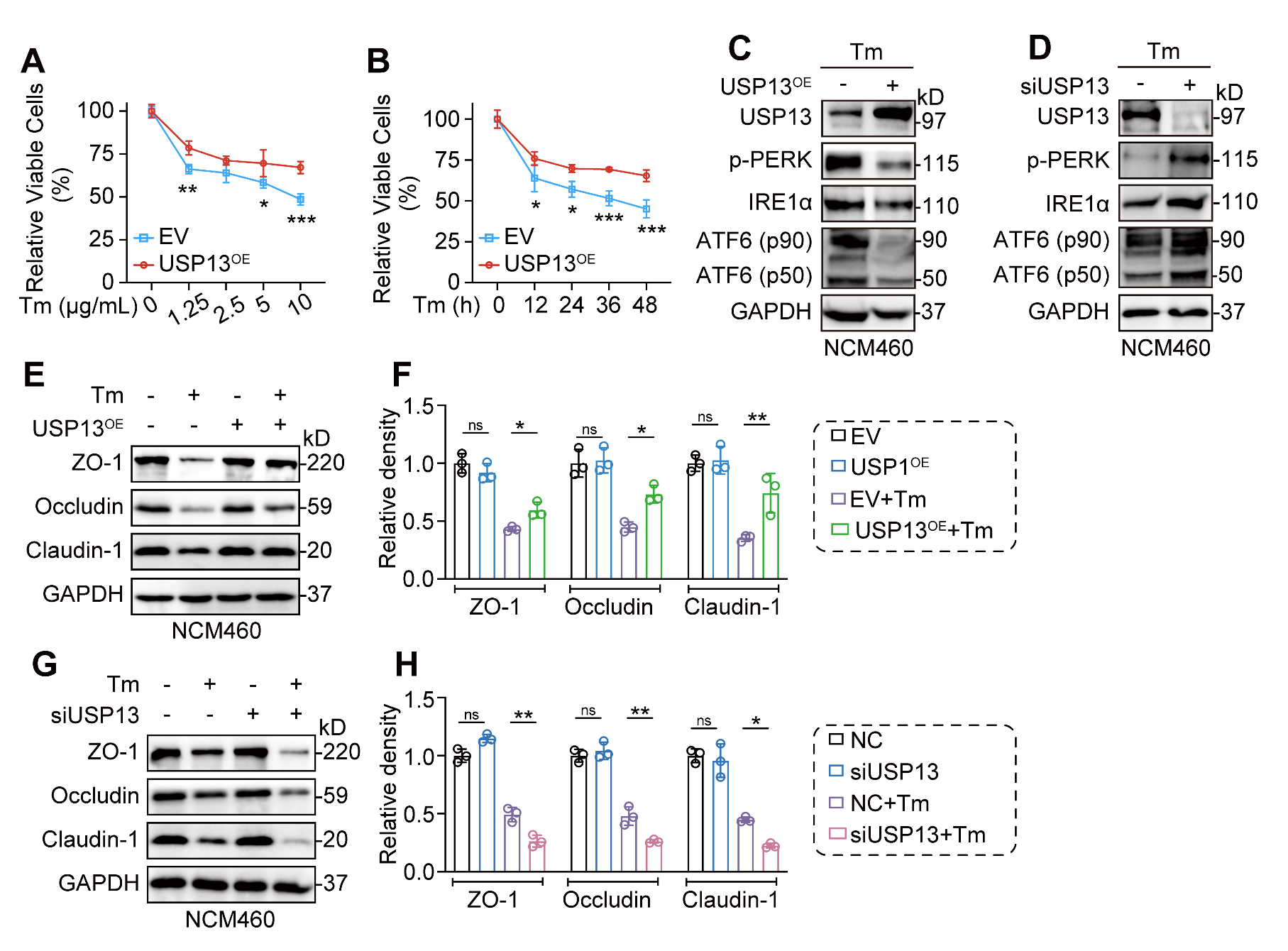
**

**Figure S3. USP13 alleviates LPS-induced ER stress and restores LPS-decreased intestinal barrier integrity. (A-B)** CCK-8 assay of viable NCM460 cells overexpressing either EV or USP13^OE^ plasmids, treated with various tunicamycin (Tm) concentrations **(A)** for 48 h or with 2.5 μg/mL Tm **(B)** for different periods (n = 3). **(C-D)** Representative immunoblots of USP13, p-PERK, IRE1α, and ATF6 p50/p90 proteins in control, USP13-overexpressing, or USP13-silenced NCM460 cells following Tm treatment (2.5 μg/mL) for 12 h. GAPDH was used as the loading control (n = 3). **(E-H)** Immunoblots of ZO-1, Occludin, and Claudin-1 proteins in control, USP13-overexpressing, or USP13-silenced NCM460 cells following Tm treatment (2.5 μg/mL) for 24 h. GAPDH was used as the loading control (n = 3). Representative blots **(G, I)** and densitometric quantification are shown **(H, J)**. Data are presented as mean ± SD. Statistical significance was determined by One-way ANOVA followed by Dunnett's multiple comparisons test. * *P* < 0.05; ** *P* < 0.01; ns = not significant.

**
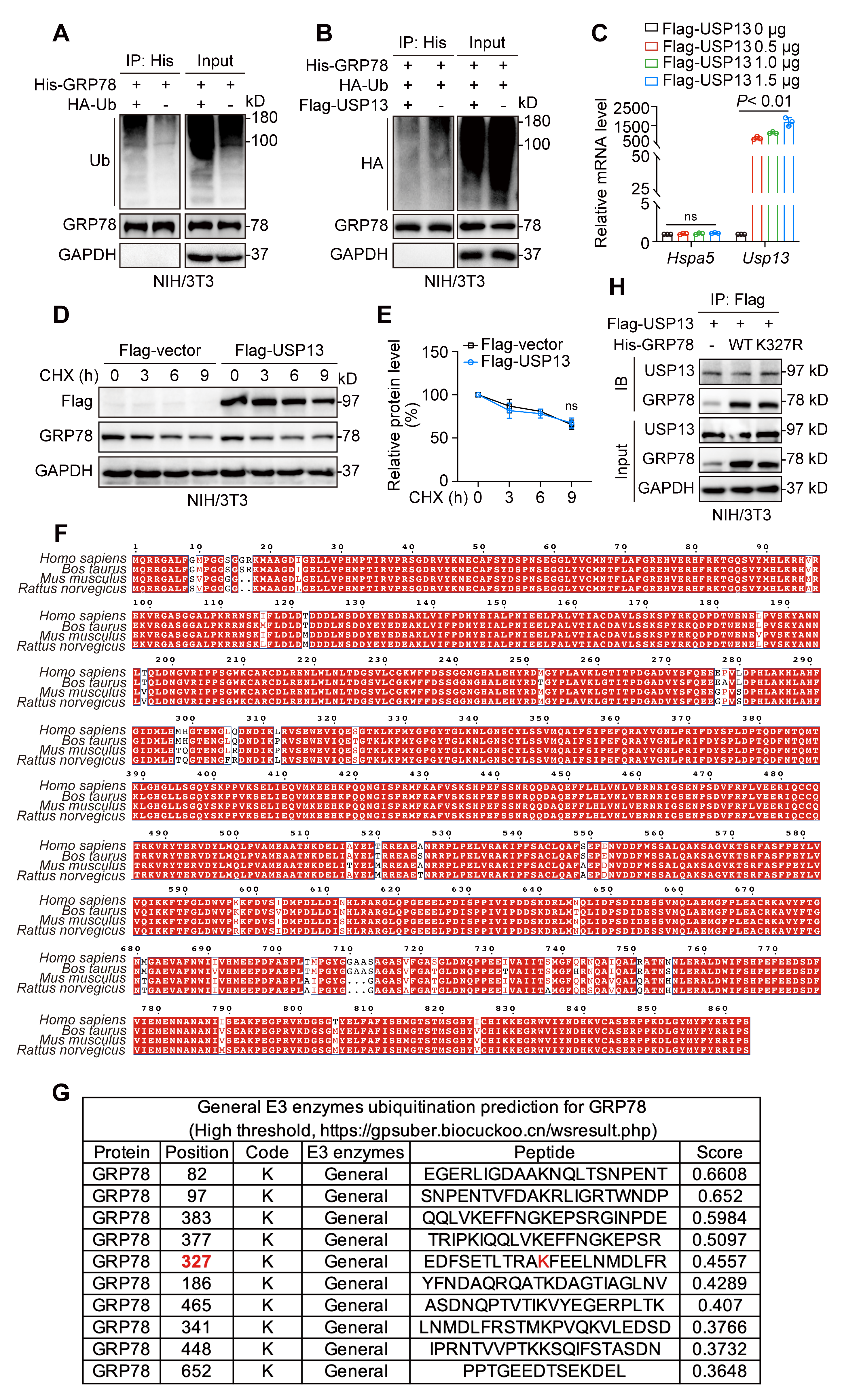
**

**Figure S4. Identification of GRP78 as the potential substrate of USP13. (A)** Co-IP of HA-Ub and His-GRP78 in lysates from NIH/3T3 cells expressing HA-Ub and His-GRP78. **(B)** Co-IP of HA-Ub and His-GRP78 in lysates from NIH/3T3 cells expressing Flag-USP13, HA-Ub, and His-GRP78. **(C)** mRNA levels of *Hspa5* and *Usp13* in lysates from NIH/3T3 cells expressing Flag-USP13. Data were normalized to the levels of *Actb* (n = 3) **(D-E)** Representative Western blot of GRP78 and Flag-USP13 in NIH/3T3 cells expressing Flag-USP13 or Flag-vector with CHX (25 μg/mL) pulse-chase stimulation **(D)** and the quantitative analysis of GRP78 (**E**; n = 3, ns = not significant). **(F)** The multiple sequence alignments of USP13 were predicted using ESpript (https://espript.ibcp.fr/ESPript/cgi-bin/ESPript.cgi). **(G)** General E3 enzymes ubiquitination prediction for GRP78. **(H)** Co-IP of Flag-USP13 and GRP78 in lysates from NIH/3T3 cells expressing Flag-USP13 and GRP78 (WT or K327R). Data are presented as mean ± SD. Statistical significance was determined by One-way ANOVA followed by Dunnett's multiple comparisons test. ns = not significant.


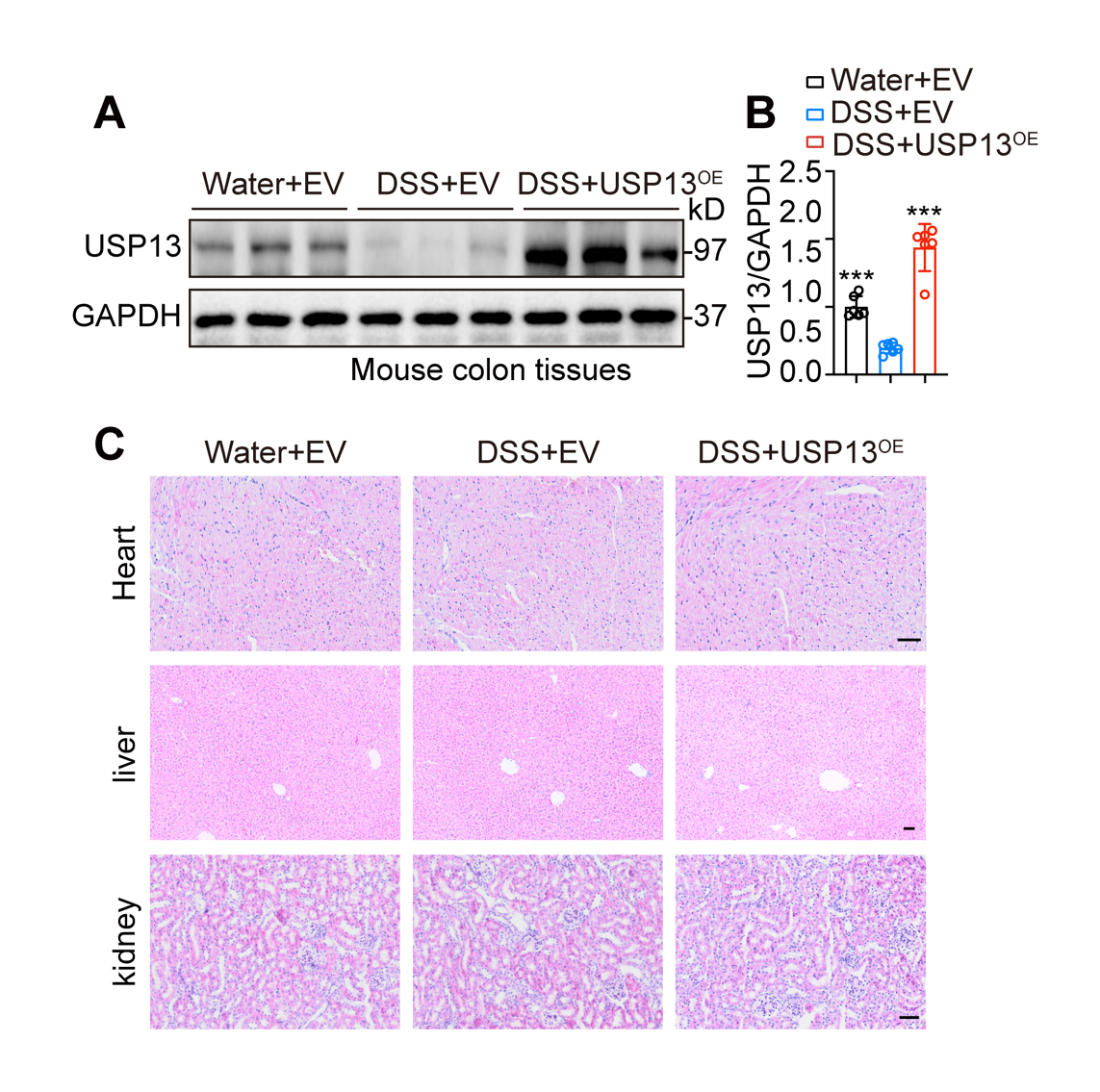


**Figure S5. (A)** Representative Western blot images of USP13 in colon tissues on day 10 after DSS initiation (n = 6). (B) Densitometric quantification for panel (A). **(C)** Representative H&E staining of the heart, liver, and kidney tissues on day 10 (scale bar: 50 μm). Data are presented as mean ± SD. Statistical significance was determined by One-way ANOVA followed by Dunnett's multiple comparisons test. *** *P* < 0.001 *vs.* DSS + EV group.
